# Topical Insulin Accelerates Wound Healing in Diabetes by Enhancing the AKT and ERK Pathways: A Double-Blind Placebo-Controlled Clinical Trial

**DOI:** 10.1371/journal.pone.0036974

**Published:** 2012-05-25

**Authors:** Maria H. M. Lima, Andréa M. Caricilli, Lélia L. de Abreu, Eliana P. Araújo, Fabiana F. Pelegrinelli, Ana C. P. Thirone, Daniela M. Tsukumo, Ana Flávia M. Pessoa, Marinilce F. dos Santos, Maria A. de Moraes, José B. C. Carvalheira, Lício A. Velloso, Mario J. A. Saad

**Affiliations:** 1 Department of Nursing, FCM, University of Campinas, Campinas, São Paulo, Brazil; 2 Department of Internal Medicine, FCM, University of Campinas, Campinas, São Paulo, Brazil; 3 Department of Cell and Developmental Biology, University of São Paulo, São Paulo, Brazil; University Hospital Hamburg-Eppendorf, Germany

## Abstract

**Background:**

Wound healing is impaired in diabetes mellitus, but the mechanisms involved in this process are virtually unknown. Proteins belonging to the insulin signaling pathway respond to insulin in the skin of rats.

**Objective:**

The purpose of this study was to investigate the regulation of the insulin signaling pathway in wound healing and skin repair of normal and diabetic rats, and, in parallel, the effect of a topical insulin cream on wound healing and on the activation of this pathway.

**Research Design and Methods:**

We investigated insulin signaling by immunoblotting during wound healing of control and diabetic animals with or without topical insulin. Diabetic patients with ulcers were randomized to receive topical insulin or placebo in a prospective, double-blind and placebo-controlled, randomized clinical trial (NCT 01295177) of wound healing.

**Results and Conclusions:**

Expression of IR, IRS-1, IRS-2, SHC, ERK, and AKT are increased in the tissue of healing wounds compared to intact skin, suggesting that the insulin signaling pathway may have an important role in this process. These pathways were attenuated in the wounded skin of diabetic rats, in parallel with an increase in the time of complete wound healing. Upon topical application of insulin cream, the wound healing time of diabetic animals was normalized, followed by a reversal of defective insulin signal transduction. In addition, the treatment also increased expression of other proteins, such as eNOS (also in bone marrow), VEGF, and SDF-1α in wounded skin. In diabetic patients, topical insulin cream markedly improved wound healing, representing an attractive and cost-free method for treating this devastating complication of diabetes.

**Trial Registration:**

ClinicalTrials.gov NCT01295177

## Introduction

Indolent, non-healing wounds constitute a major problem that plagues those with diabetes. Approximately 15% of all patients with diabetes will, at some time, have non-healing wounds, despite insulin treatment and a meticulously-controlled diet, and this is the leading cause of lower extremity amputation [1], [2]. It is well known that the basic cellular and molecular mechanisms that result in wound healing involve cell adhesion, migration, proliferation, differentiation, and apoptosis [2]. Abnormalities of distinct factors contribute to defective wound healing in diabetes, including decreased growth factor production [3], angiogenic response [4], [5], macrophage function [4], collagen accumulation, epidermal barrier function, and keratinocyte and fibroblast migration and proliferation [2].

Absolute or relative lack of insulin or insulin action is a hallmark of diabetes, and defective insulin action in the skin has been proposed as an important mechanism contributing to wound healing defects in this disease [6]. Previous data, although not well controlled, showed that topical insulin accelerates wound healing in the skin of diabetic rats and humans [7], [8], [9], [10], [11], [12], [13], [14], [15], but in these studies no mechanism for this insulin effect was proposed or investigated. It is known that insulin stimulates the growth and development of different cell types, and affects proliferation, migration, and secretion by keratinocytes, endothelial cells, and fibroblasts [12], [16], [17], [18], [19]. At least part of the effects of insulin in the skin may be via canonical signal transduction, as previously shown [20], and we suspect that upon reconstitution of normal insulin signaling in the wounded skin of diabetic subjects, healing may be corrected.

The purpose of this study was to investigate the regulation of the insulin signaling pathways in wound healing and skin repair of normal and diabetic rats and, in parallel, the effect of an insulin cream on wound healing in these pathways. Since results in experimental animals were very promising, we also performed a pilot study employing this insulin cream in a prospective, double-blind and placebo-controlled, randomized clinical trial of wound healing in diabetic patients.

## Materials and Methods

### Materials

Anti-phosphotyrosine (αPY), anti-insulin receptor substrate (IRS)-1, anti-IRS-2, anti- Src homology 2/α-collagen-related (SHC), anti-phospho-extracellular signal-regulated protein kinase (ERK)-1/2, anti-ERK1/2, anti-endothelial nitric oxide synthase (eNOS), anti-phospho- eNOS, anti-glycogen synthase kinase (GSK3), anti-phospho-GSK3, anti-serine-threonine kinase (AKT), anti-stromal cell-derived factor (SDF) 1α, anti-vascular endothelial growth factor (VEGF), anti-β-actin, and anti-goat- and anti-rabbit IgG-peroxidase-conjugated antibodies were from Santa Cruz Technology (Santa Cruz, CA, USA). Anti-phospho-AKT (serine 473) antibody was from Cell Signaling Technology (Beverly, MA, USA). Routine reagents were purchased from Sigma Chemical Co. (St. Louis, MO, USA) unless specified elsewhere. Protein A was from Amersham (Buckinghamshire, UK). Materials for immunostaining were from Vector Laboratories Inc. (Burlingame, CA, USA).

### Animals

Male Wistar rats were provided by the University of Campinas Central Breeding Center. Six-week-old male rats were divided into six groups: 20 control rats with intact skin (CC); 20 control rats submitted to a skin excision wound (WC); 20 control rats submitted to a skin excision wound and treated with topical insulin cream (WCI); 20 rats treated with streptozotocin (STZ) to induce diabetes (DD); 20 STZ-induced diabetic rats submitted, after four-seven days, to a skin excision wound (WD); and 20 STZ-induced diabetic rats submitted, after four-seven days, to a skin excision wound and treated with topical insulin cream (WDI). All groups received standard rodent chow and water *ad libitum*. This study was approved by the Ethical Committee for Animal Use of the University of Campinas (ID protocol: 1941-1) The approval is available as supporting information; see Approval S1.

### Skin excision wound and use of insulin cream

Four groups of animals were submitted to only one skin excision wound per animal (WC, WCI, WD, and WDI). Wounding was performed under general anesthesia induced by sodium amobarbital (15 mg/kg body weight, i.p.), and the animals were used 10–15 min later, i.e., as soon as anesthesia was assured by the loss of pedal and corneal reflexes. After shaving the dorsum, a full-thickness excision wound (4.0×4.0 mm) was made to the level of the epidermis and dermis. The wound was not sutured or covered and healed by secondary intention. Collagenase production is most prominent at days three and five post-wounding [21], and the appearance of fibroblasts and the subsequent deposition of extracellular matrix components such as collagen, elastin, glycoproteins, and fibronectin seems to be present 24 hours after wounding, reaching a maximal amount after 5–6 days, followed by a gradual decrease after nine days [22]. Fibroblasts in the granulation tissue of excision wounds are also observed after three days [23], [24]. The excision skin wound was evaluated clinically every day, and rats were used for experiments after four or eight days, according to the protocol specified in each experiment. The insulin cream used was prepared with regular insulin (0.5 U/g cream) in the pharmacy of our University Hospital and holds the patent number, PI 0705370-3 (University of Campinas, Brazil). In preliminary experiments, we used different concentrations of insulin to prepare the cream (0.0, 0.1, 0.25, 0.5, and 1.0 U/100 g), but the doses that induced the best effect in wound healing were 0.5 U and 1.0 U/100 g. The dose of 1.0 U/100 g, in some animals, induced alterations in plasma glucose. Therefore, we used a concentration of 0.5 U/100 g for all experiments

The cream under study—placebo or with insulin—was applied locally to cover the excision immediately after wounding (day 0) and re-applied daily until the end of the experiment (day 4 or day 8). The excision wound of the diabetic animals received placebo (WD) or the cream with insulin (WDI).

### STZ treatment

Overnight-fasted rats were rendered diabetic by a single intraperitoneal injection of STZ (Sigma; 100 mg/Kg in citric buffer, pH 4.5) [23]. Control groups received an equivalent volume of citric buffer, pH 4.5. Rats were used in the experiments between four and seven days after receiving STZ injection, when blood glucose reached stable levels over 300 mg/dL [24]. Plasma glucose levels were determined by the glucose oxidase method using blood samples collected from the animal tail before the experiments were performed.

### Tissue extraction and immunoblotting

Rats from each group were anesthetized with sodium amobarbital (15 mg/kg body weight, i.p.) and were used 10–15 min later, i.e., as soon as anesthesia was assured by the loss of pedal and corneal reflexes. For evaluation of protein expression and activation of signal transduction pathways, the skin wound of anesthetized rats was excised and immediately homogenized in extraction buffer (1% Triton-X 100, 100 mM Tris, pH 7.4, containing 100 mM sodium pyrophosphate, 100 mM sodium fluoride, 10 mM EDTA, 10 mM sodium orthovanadate, 2 mM PMSF, and 0.1 mg of aprotinin/ml) at 4°C with a Polytron PTA 20S generator (Brinkmann Instruments model PT 10/35) operated at maximum speed for 30 sec. The extracts were centrifuged at 15,000 rpm at 4°C in a Beckman 70.1 Ti rotor (Palo Alto, CA) for 45 min to remove insoluble material, and the supernatant of these tissues was used for immunoblotting with antibodies against IR (1∶1000), IRS-1 (1∶100), IRS-2 (1∶100), phospho-AKT (1∶1000), AKT (1∶1000), phospho-ERK (1∶1000), ERK (1∶1000), phospho-GSK3 (1∶1000), GSK3 (1∶100), phospho-eNOS (1∶100), eNOS (1∶100), SHC (1∶1000), VEGF-1 (1∶1000), SDF-1α (1∶200), and SHC (1∶1000). Whole tissue extracts from all animals were mixed with Laemmli buffer and similar-sized aliquots (20 µg protein) were subjected to SDS-PAGE. Following transfer to nitrocellulose, blots were probed with the antibodies described above. The blots were subsequently incubated with peroxidase-conjugated antibodies (anti-rabbit or anti-goat). The excision of wounds for tissue extraction and immunoblotting was performed on day 4 after the incision, unless specified elsewhere.

### Use of inhibitors of phosphatidyl-inositol 3-kinase (PI3K), LY294002, and/or of mitogen-activated protein kinase/extracellular-signal-regulated kinases (MAPK/ERK), PD98059

In order to evaluate the relevance of the PI3K and MAPK pathways in the wound healing of diabetic rats, we treated these animals on day 6 after beginning the use of the insulin cream. Therefore, there were seven groups of diabetic rats: wounded rats, wounded rats treated with LY94002, wounded rats treated with PD98059, wounded rats treated with insulin cream, wounded rats treated with LY94002 and insulin cream, wounded rats treated with PD98059 and insulin cream, and wounded rats treated with LY94002, PD98059 and insulin cream.

### Histology and morphometrical analysis

Skin wounds from 3–4 wounded diabetic rats treated with placebo cream (WD) and wounded diabetic rats treated with insulin cream (WDI), on the 4^th^ and 8^th^ days after experimental wounding, were excised and processed for morphological analysis. Samples were fixed in 4% formaldehyde solution for 8 h at room temperature and processed for Paraplast® embedding. Transversal 7 µm-thick sections were stained with hematoxylin and eosin (HE). For morphological analysis of the wounds (HE staining), the tissue was observed using a ×10 objective. Data were compared by ANOVA and Tukey's post-test (p<0.05).

### Clinical Protocol

**Figure 1:**
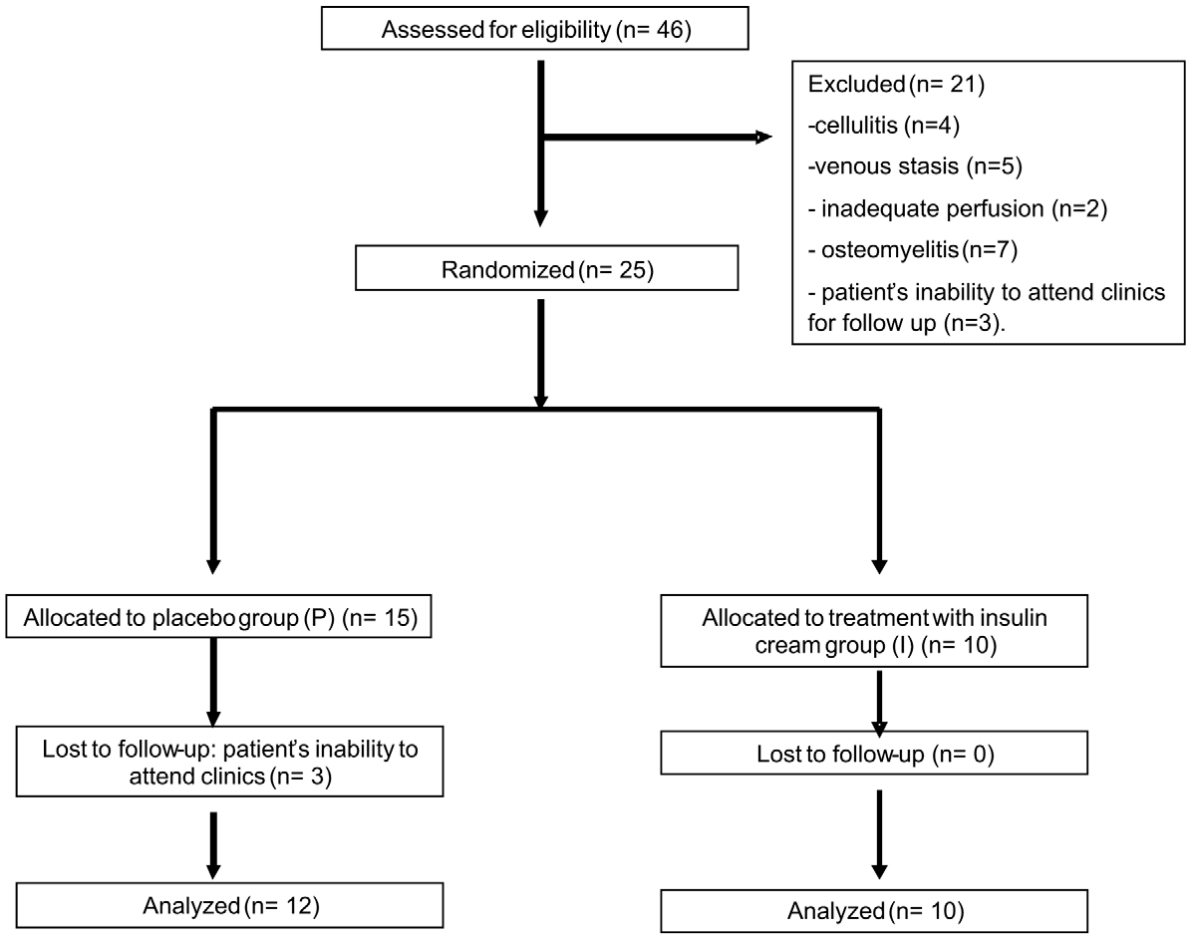
Flowchart.

The protocol for this trial and supporting CONSORT checklist are available as supporting information; see Checklist S1 and Protocol S1. This study was double-blind and placebo-controlled in design, and conducted in the State University of Campinas, Brazil, from January 2004 to September 2007. Eligible patients were between 18 and 80 years old, both male and female individuals with type 1 or type 2 diabetes mellitus, and with wounds that had not healed for at least three months, with serum creatinine below 1.5 mg/dL. Patients were excluded: if they did not satisfy the inclusion criteria; if the wounds were infected; if cellulites, venous stasis, inadequate perfusion, or osteomyelitis were present; or if the patients were unable to attend the follow-up. The study was conducted in accordance with the guidelines of the University of Campinas for clinical trials and the Declaration of Helsinki. We also obtained ethics approval for the human study from the University Hospital of the State University of Campinas, where participants were recruited and human experimentation was conducted (Clinical Trials identifier: NCT01295177); The approval is available as supporting information; see Approval S2. Diabetic patients with chronic foot ulcers for at least three months were selected for this study with ulcer grade 1 and 2 according to the Wagner classification [25], with significant soft tissue defects of the feet. All patients had undergone debridement for the ulcer, followed by standard moist gauze treatment with 0.9% normal saline for at least four weeks, resulting in no more than a 15% reduction in ulcer dimensions. All target wound surfaces were ≥2.0 cm and ≤18 cm in any one dimension after debridement. Peripheral neuropathy was evaluated by two methods: monofilament testing using a Semmes-Weinstein 5.07 (10 g) monofilament and the base of a vibrating tuning fork (C 128 Hz). From January 2004 to September 2007, 46 patients were assessed for eligibility for this study. However, 21 patients were excluded due to at least one of the following exclusion criteria: presence of cellulitis (n = 4), venous stasis (n = 5), inadequate perfusion (n = 2), osteomyelitis (n = 7), and patient's inability to attend clinics for follow up (n = 3). All patients included in the study provided a written informed consent. Twenty-five patients were initially enrolled; however, three of them did not succeed in completing the protocol due to inability to attend the clinic (Fig. 1). All patients included in this study had type 2 diabetes: 11 patients used subcutaneous insulin and oral anti-diabetic drugs, and 11 patients used only oral anti-diabetic drugs. Initial laboratory analysis consisted of leukocyte and platelet count, hemoglobin, glucose, creatinine, and glycosylated hemoglobin. During the follow-up, only glucose and glycosylated hemoglobin were evaluated. The patients were randomly assigned to receive treatment with placebo cream (cream containing vehicle but without insulin; group P), or insulin cream (group I) for eight weeks. The patients were instructed to clean their wounds with sterile gauze and 0.9% sterile saline daily prior to assessment, putting the cream (placebo or insulin) and the dressing on afterwards. All wounds were photographed digitally at the beginning of the study and then at least every other week during the weekly visits of the patients to the hospital. Computerized planimetry was used (Texas Health Science Center at San Antonio ImageTool, version 3.0, as downloaded from www.ddsdx.uthsca.edu/dig/itdesc.html) to compare the progression of wound healing in the two groups.

### Statistical Analysis

Wound dimensions were calculated in a blinded fashion and analyzed for homogeneity and significance using SPSS, version 13.0 (SPSS, Inc., Chicago, IL, USA). All continuous variables are expressed as means ± SE. One-way analysis of variance (ANOVA) was used to assess the differences in a continuous variable between the two groups of patients, and the three or four groups of animals, using Bonferroni post-test. Post hoc analysis was performed using Tukey's test for the histology analysis. All tests were two-tailed, and the level of significance employed was *P*<0.05.

## Results

### Time-course of expression of insulin signaling proteins in the wounded skin of rats

**Figure 2:**
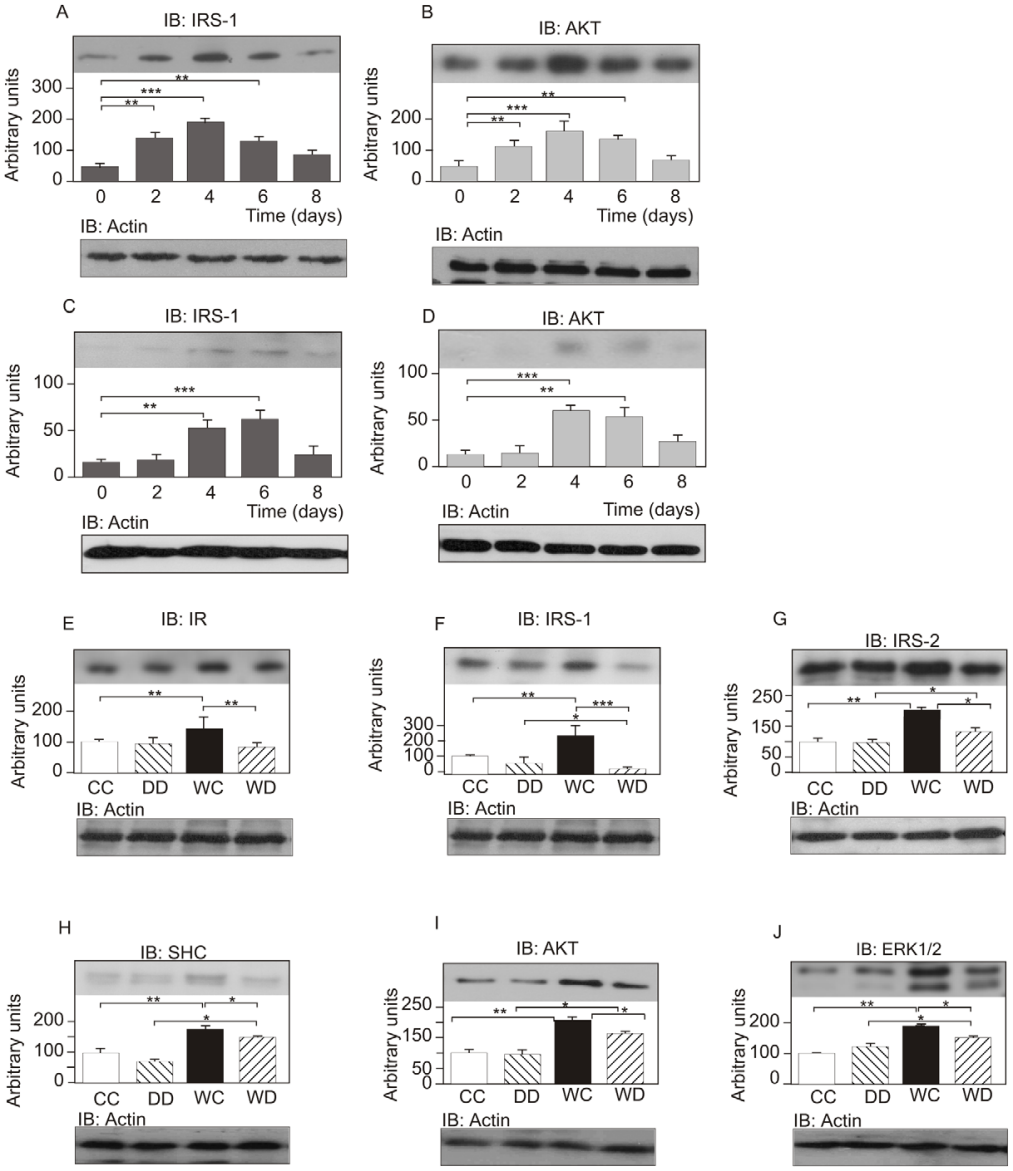
Time-course of IRS-1 and AKT expression following skin wounding in control (A, B) and diabetic animals (C, D). Tissue protein levels in the intact skin of control (CC) and intact skin of diabetic (DD) rats and in the wounded skin of control (WC) and wounded skin of diabetic animals (WD). Skin and wound extracts from control and diabetic rats were prepared, as described in *Materials and Methods*, four days after the wound incision. Tissue extracts were immunoblotted with (E) anti-IR antibody, (F) anti-IRS-1 antibody, (G) anti-IRS-2 antibody, (H) anti-SHC antibody, (I) anti-AKT antibody, and (J) anti-ERK1/2 antibody. Equal protein loading was confirmed by reblotting the membranes with anti-β-actin. Data were compared by ANOVA and Bonferroni post-test, and represented by the mean and standard deviation for each group of scanning densitometry of six different animals per group. *P<0.05 between groups. The Fig. 2G IB: Actin panel presents material previously published in https://doi.org/10.2337/db11-0390, and is excluded from this article's copyright license.

Tissue extracts from the excision wounds were obtained at 0, 2, 4, 6, and 8 days after the initial wounding incision, and were used for immunoblotting with anti-IRS-1 and anti-AKT antibodies, in order to determine the effect of wound healing on the level of these proteins in the skin of control rats. Results showed that there is a consistent increase in both proteins two days after the initial wound excision, reaching a maximum on day 4, and then decreasing to levels similar to baseline at day 8, when most wounds were completely healed (Fig. 2A and 2B). In the skin of diabetic rats, results followed a similar time-course, but the increases in the protein levels were much less evident on each day, and on day 8 the wound had not yet healed (Fig. 2C and 2D). In further experiments, day 4 was used to compare the levels of proteins involved in the early steps of insulin action between wound healing in the skin of diabetic and control rats.

### Insulin signaling proteins in wounded skin of control and diabetic rats

An increase in the IR protein level was observed in the wounded skin of rats, compared to control rats with intact skin (WC = 143±25% *vs.* CC = 100±8%; P<0.05; Fig. 2E). IR protein levels were lower in the wounded skin of STZ-diabetic rats compared to the wounded control rats (WC = 143±25% *vs.* WD = 98±11%; P<0.05; Fig. 2E). In the wounded skin of control rats, there was an increase in IRS-1 levels, compared to the intact skin of control rats (WC = 241±57% *vs.* CC = 100±6%; P<0.05; Fig. 2F). IRS-1 protein levels were decreased in the wounded skin of diabetic rats, compared to the wounded skin of control rats (WD = 27±13% *vs.* WC = 241±57%; P<0.05; Fig. 2F) and intact skin of diabetic rats (WD = 27±13% *vs.* DD = 78±18%; P<0.05; Fig. 2F). When blots were probed with anti-IRS-2 antibody, we observed an increase in the protein levels of IRS-2 in the wounded skin of control rats, compared to the intact skin of control animals (WC = 202±17% *vs.* CC = 100±15%; P<0.05; Fig. 2G). In the wounded skin of diabetic rats, IRS-2 protein levels were higher than in the intact skin of diabetic rats (WD = 131±14% *vs.* DD = 97±17%; P<0.05; Fig. 2G), but lower than the wounded skin of control rats (WD = 131±14% *vs.* WC = 202±17%; P<0.05; Fig. 2G). SHC protein levels were increased in the wounded skin of control rats compared to the intact skin of control animals (WC = 178±11% *vs.* CC = 100±12%; P<0.05; Fig. 2H). SHC protein levels were decreased in the wounded skin of diabetic rats, compared to the wounded skin of control rats (WC = 178±11% *vs.* WD = 151±9%; P<0.05; Fig. 2H), but increased compared to the intact skin of diabetic rats (WD = 151±9% *vs.* DD = 75±7%; P<0.05; Fig. 2H). When membranes were probed with anti-AKT antibody, the expression of this protein was increased in the wounded skin of control rats, compared to the intact skin of control animals (WC = 208±10% *vs.* CC = 100±10%; P<0.05; Fig. 2I). AKT protein levels were decreased in the wounded skin of diabetic rats compared to the wounded skin of control rats (WD = 165±8% *vs.* WC = 208±10%; P<0.05; Fig. 2I), but increased compared to the intact skin of diabetic rats (WD = 165±8% *vs.* DD = 168±12%; P<0.05; Fig. 2I). ERK1/2 protein levels were increased in the wounded skin of control rats, compared to the intact skin of control animals (WC = 189±6% *vs.* CC = 100±3%; P<0.05; Fig. 2J), but they were decreased in the wounded skin of diabetic rats when compared to the wounded skin of control rats (WD = 152±5% *vs.* WC = 189±6%; P<0.05; Fig. 2J) and increased when compared to the intact skin of diabetic rats (WD = 152±5% *vs.* DD = 118±14%; P<0.05; Fig. 2J).

### Effect of a topical insulin cream on insulin signaling proteins in wounded skin

**Figure 3:**
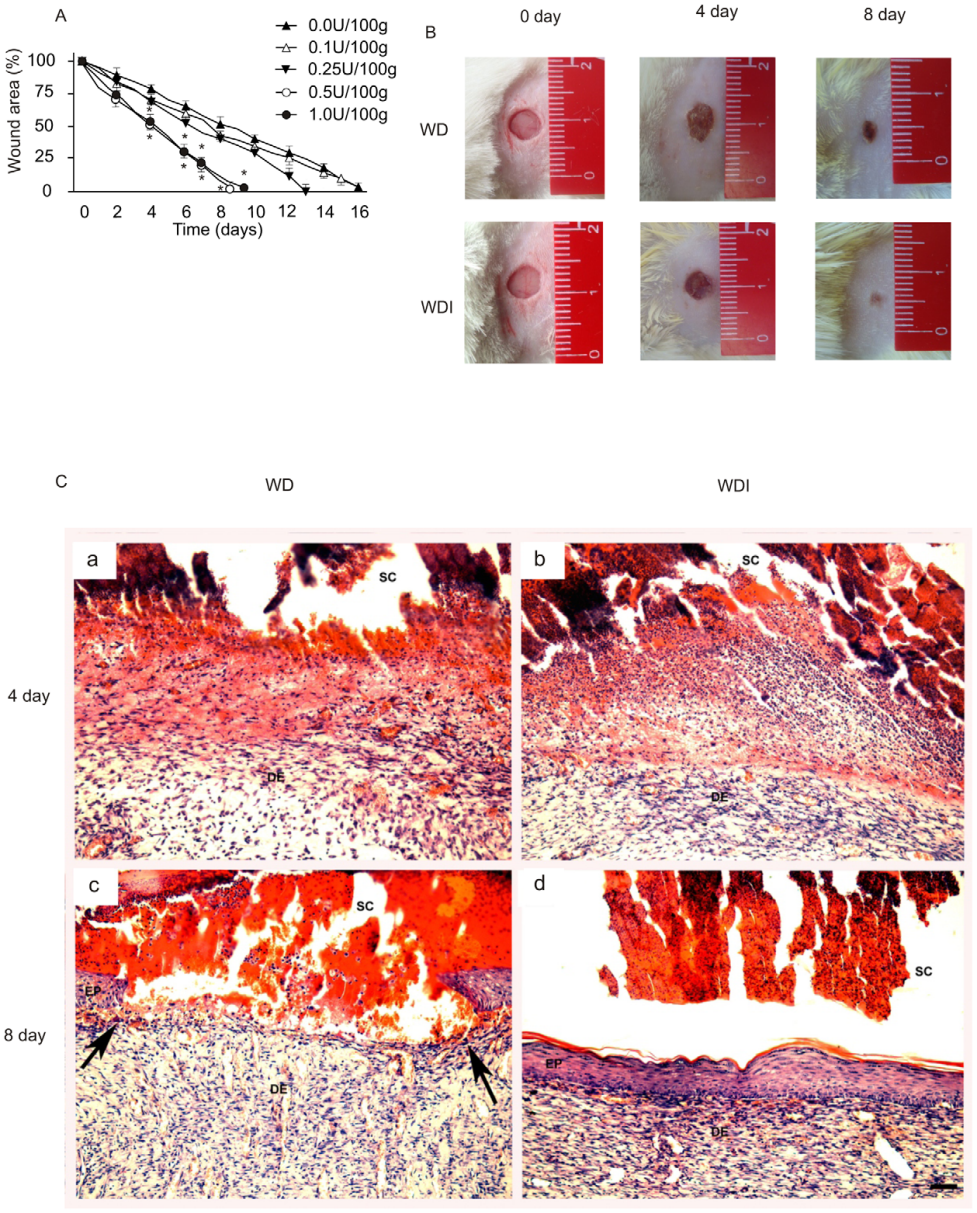
Macroscopic wound closure in diabetic rats treated with different concentrations of insulin cream. Representative wound area values during the time-course of 16 days. Values are expressed as mean ± SEM of at least 10 animals per group. * p<0.05 comparing 0.5 U and 1.0 U *vs.* 0.0 U, 0.25 U, and 0.1 U of insulin/100 g of cream. (A) Photos showing the time-course of wound healing in rats 0, 4, and 8 days after they received the insulin cream. (B) Morphology of the wounds after four days (a–b) and eight days (c–d) in rats treated with WD cream (left column - a, c) or rats treated with WDI cream (right column - b, c). Arrowheads in c show the extremities of the unclosed wound, while d shows complete closure of the epithelium. EP = epidermis, DE = dermis, SC = scab. Magnification bar in D = 50 µm.

In order to establish the dose of insulin of the cream, we performed a dose-course experiment in diabetic rats, with the following concentrations of insulin: 0.0, 0.1, 0.25, 0.5, and 1.0 U/100 g of cream. Wounds were treated with the insulin cream and measured daily. We observed that insulin concentrations of 0.5 U and 1.0/100 g presented the best wound healing rate (Fig. 3A). The dose of 1.0 U/100 g, in some animals, induced alterations in plasma glucose, and therefore, we used a concentration of 0.5 U/100 g for all experiments.

We next investigated the effect of an insulin cream on the wound healing of diabetic rats (Fig. 3B). The effectiveness of the topical insulin cream treatment in accelerating healing could be observed in HE-stained sections. Four days after wounding, we observed the presence of a scab containing many inflammatory cells, which were mostly neutrophils. The connective tissue of the dermis underneath this scab contained many lymphocytes and plasma cells. After eight days of wounding, the wound had closed in all animals treated with WDI; the epidermis was completely reconstituted, even when a remaining scab was still present at the wound surface, although skin appendages were absent (Fig. 3C). The dermis was better organized concerning cells and collagen fibers arrangement. However, at this stage WD animals did not have a complete wound closure and keratinocytes were still migrating to close the wound (Fig. 3C). The dermis was much less organized than the WDI group.

**Figure 4:**
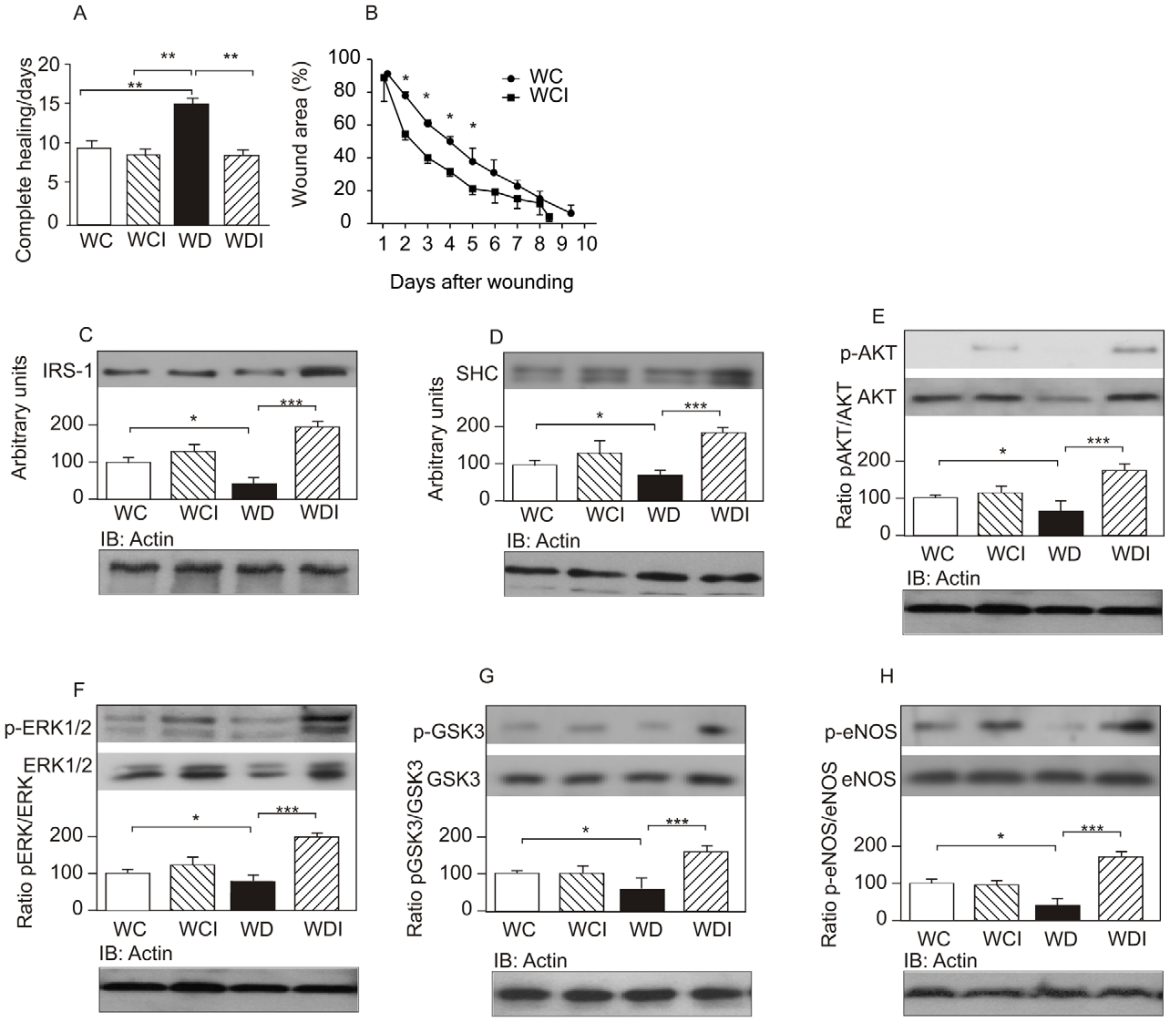
Days to achieve complete healing in wounded control rats (WC), wounded control rats that received the insulin cream (WCI), wounded diabetic rats (WD), and wounded diabetic rats that received insulin cream (WDI). (A) Wound area was quantified every day and expressed as the percentage of the original wound area. (B) Wound extracts were prepared as described in*Materials and Methods* four days after the wound incision and were immunoblotted with (C) anti-IRS-1, (D) anti-SHC, (E) anti-phospho-AKT, (F) anti-phospho-ERK1/2, (G) anti-phospho-GSK3, and (H) anti-phospho-eNOS antibodies. To determine the protein levels of AKT, ERK1/2, GSK3, and eNOS, the membranes were stripped and reprobed with anti-AKT, -ERK1/2, -GSK3 and -eNOS. Equal protein loading was confirmed by reblotting the membranes with anti-β-actin. Data were compared by ANOVA and Bonferroni post-test, and represented by the mean and standard deviation for each group of scanning densitometry of six different animals per group, and the bar graphs represent the ratio of phosphorylation/protein. *p<0.05 between groups; **p<0.05 *vs.* WCI.

It is important to mention that the use of insulin cream did not induce changes in blood glucose levels of control or diabetic animals (Table 1). Results showed that when similar incisions are performed in control and diabetic rats, the mean healing time is nine days for controls and 15 days for diabetic animals. Therefore, the control animals had a 40% increase in the wound healing time compared to diabetic animals (Fig. 4A). However, when the topical cream with insulin was used on the wound, the mean healing time in diabetic animals was similar to that of controls (Fig. 4A). Notably, the time to complete the healing process in control rats was unaffected by the topical insulin cream. However, the percentage of closure showed a difference in the first six days. Our data showed that the wound area of control rats treated with insulin cream significantly decreased at several time-points, in accordance with previous data [9]. We showed that by day 2 and 4, the decrease in wound area induced by insulin was greater than in the placebo (WCI: 38.27±1.5% *vs.* WC: 22±0.45% on day 2, p<0.05; WCI: 68.64±1.2% *vs.* WC: 49.75±1.71% on day 4, p<0.05). However, although the time to closure was decreased in control animals treated with insulin, the difference was not statistically significant (WCI: 8.4±0.91 days *vs.* WC: 9.3±1.4 days, p = 0.123; Fig. 4B).

**Table 1:** Plasma glucose levels of 10 diabetic and 10 control animals that received the cream with insulin or with placebo.

Time after treatment	WC	WCI	WD	WDI
1 h	93±5	98±2	465±26	492±26
3 h	91±4	98±3	502±18	523±20
6 h	97±3	99±3	528±24	481±30
48 h	91±7	95±2	480±30	478±35
96 h	92±6	97±3	461±25	482±30

The values are represented as the mean ± SEM of 10 experiments. WC: wounded control rats; WCI: wounded control rats treated with insulin cream; WD: diabetic animals treated with placebo cream; WDI: diabetic rats treated with insulin cream. P<0.05 between control and diabetic rats.

The effect of insulin cream was also investigated in the proteins involved in insulin signaling. Results showed that the blunted increase in IRS-1, SHC, AKT, and ERK1/2 observed in diabetic animals, was completely reversed after the use of the cream (Fig. 4C–F). Downstream of AKT, two signaling proteins are important for wound healing: GSK3β and eNOS. We also investigated the regulation of these proteins in the wound healing of diabetic animals. Results showed that there was a significant decrease in GSK3β and eNOS protein levels in the wounded skin of diabetic animals to 55±6% and 46±8% compared to the wounded non-diabetic control rats, respectively, and these levels were completely reversed after topical administration of the insulin cream (Fig. 4G–H).

### Effect of insulin cream with or without inhibitors of PI3K/AKT and/or MAPK/ERK pathways on wound healing of diabetic rats

**Figure 5:**
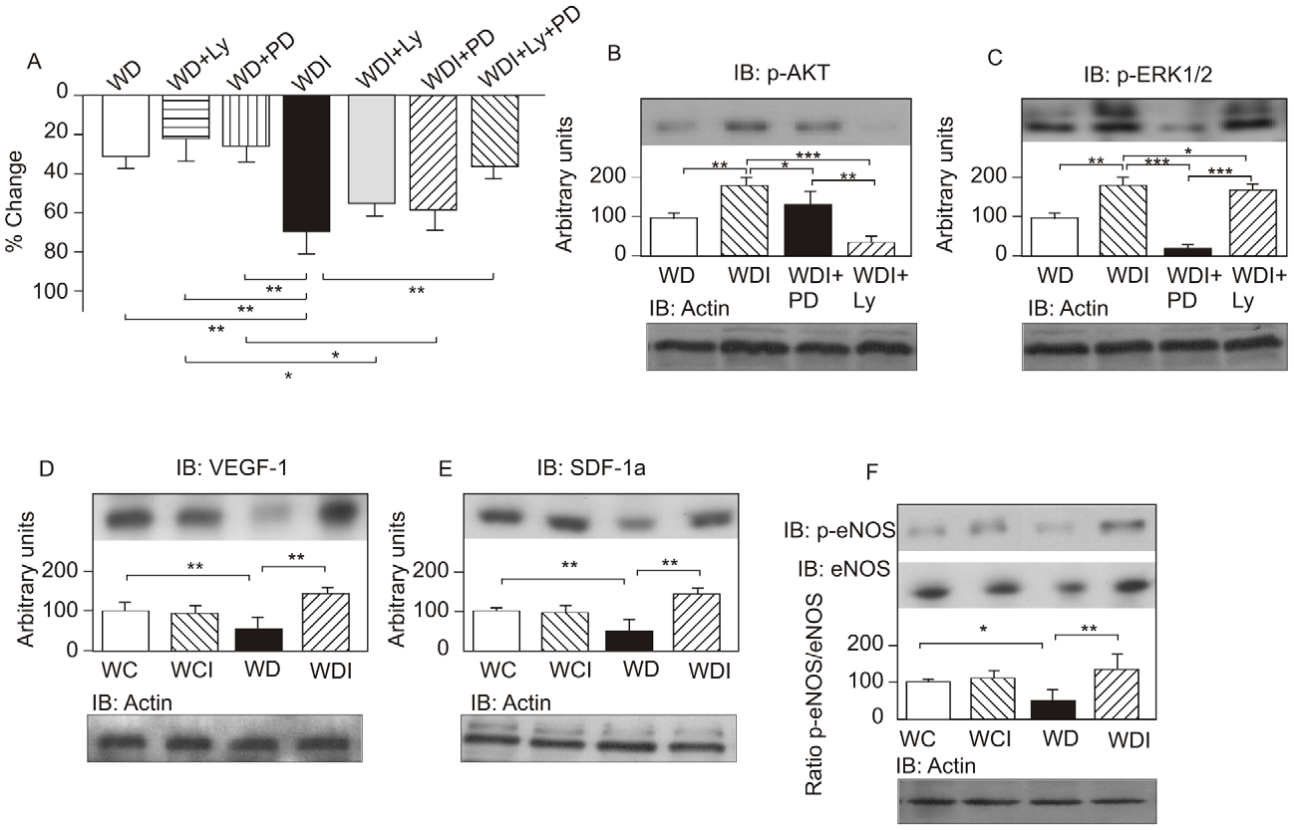
Effect of the inhibitors of PI3K and of ERK. Effect of inhibitors of PI3K (LY294002) and/or ERK (PD98059) on wound healing on day six after beginning use of the topical cream, represented as a percentage of change in wound area in wounded diabetic rats (WD), wounded diabetic rats treated with LY294002 (WD+Ly), wounded diabetic rats treated with PD98059 (WD+PD), wounded diabetic rats treated with insulin (WDI), wounded diabetic rats treated with insulin and Ly (WD+Ly), wounded diabetic rats treated with insulin and PD (WD+PD), and wounded diabetic rats treated with insulin, Ly and PD (WDI+Ly+PD). (A) AKT phosphorylation in wound healing. (B) ERK1/2 phosphorylation in wound healing. (C) VEGF-1 protein expression in wound healing. (D) SDF-1α protein expression in wound healing. (E) eNOS phosphorylation and eNOS protein expression in wound healing. (F) Equal protein loading was confirmed by reblotting the membranes with anti-β-actin. Data were compared by ANOVA and Bonferroni post-test, and represented by the mean and standard deviation for each group of scanning densitometry of six different animals per group. *P<0.05 between groups; **P<0.05 *vs.* WD *vs.* WD+Ly *vs.* WD+PD; ***P<0.05 *vs.* WDI.

Since our data show an increase in PI3K/AKT and in the MAPK/ERK pathway, we next investigated the effect of inhibitors of these pathways during use of the insulin cream for wound healing (Fig. 5A–C). The results show that the use of either the inhibitor of PI3K (LY294002) or of MAPK (PD98059), together with insulin cream, reduced the rate of wound healing by ∼20%, compared to animals treated with insulin cream alone. It is relevant to mention that the families commonly referred to as ERKs are activated by parallel protein kinases cascades, named MAPKs [26], [27]. These data suggest that insulin uses both proteins to improve wound healing. In this regard, the simultaneous use of the two inhibitors in the insulin cream almost completely abolished the effect of the insulin cream. The treatment with LY294002 led to an impairment of the phosphorylation of AKT (Fig. 5B), a downstream protein of the PI-3K activation, and the treatment with PD98059 led to the impairment of the phosphorylation of ERK (Fig. 5C), suggesting that these inhibitors were effective. The use of these inhibitors in wounded diabetic rats treated with placebo cream also led to a trend towards decreasing wound healing rate, although without statistical significance, reinforcing the data that the pathways PI3K and ERK are involved in the wound healing process stimulated by the insulin cream.

### Effect of insulin cream on eNOS in bone marrow and on VEGF and SDF-1α in wound healing in diabetic rats

It has recently been shown that an increase in the migration of endothelial progenitor cells (EPCs) from bone marrow to wounded skin is an essential step in wound healing [12], [16]. The release of EPCs involves activation of eNOS in the bone marrow by VEGF, which is produced in wounded skin, enhancing the mobilization of EPCs, which are recruited to the skin wound site by an increase in tissue levels of SDF-1α. We therefore investigated the effect of the insulin cream on the regulation of this process. Results show that in the wounded skin of diabetic animals, there were decreases in VEGF and SDF-1α, and in bone marrow there was also a decrease in eNOS phosphorylation. These alterations were completely reversed by topical administration of an insulin cream in diabetic animals (Fig. 5D–F).

### Effect of the topical insulin cream on wound healing in the skin of diabetic patients

**Figure 6:**
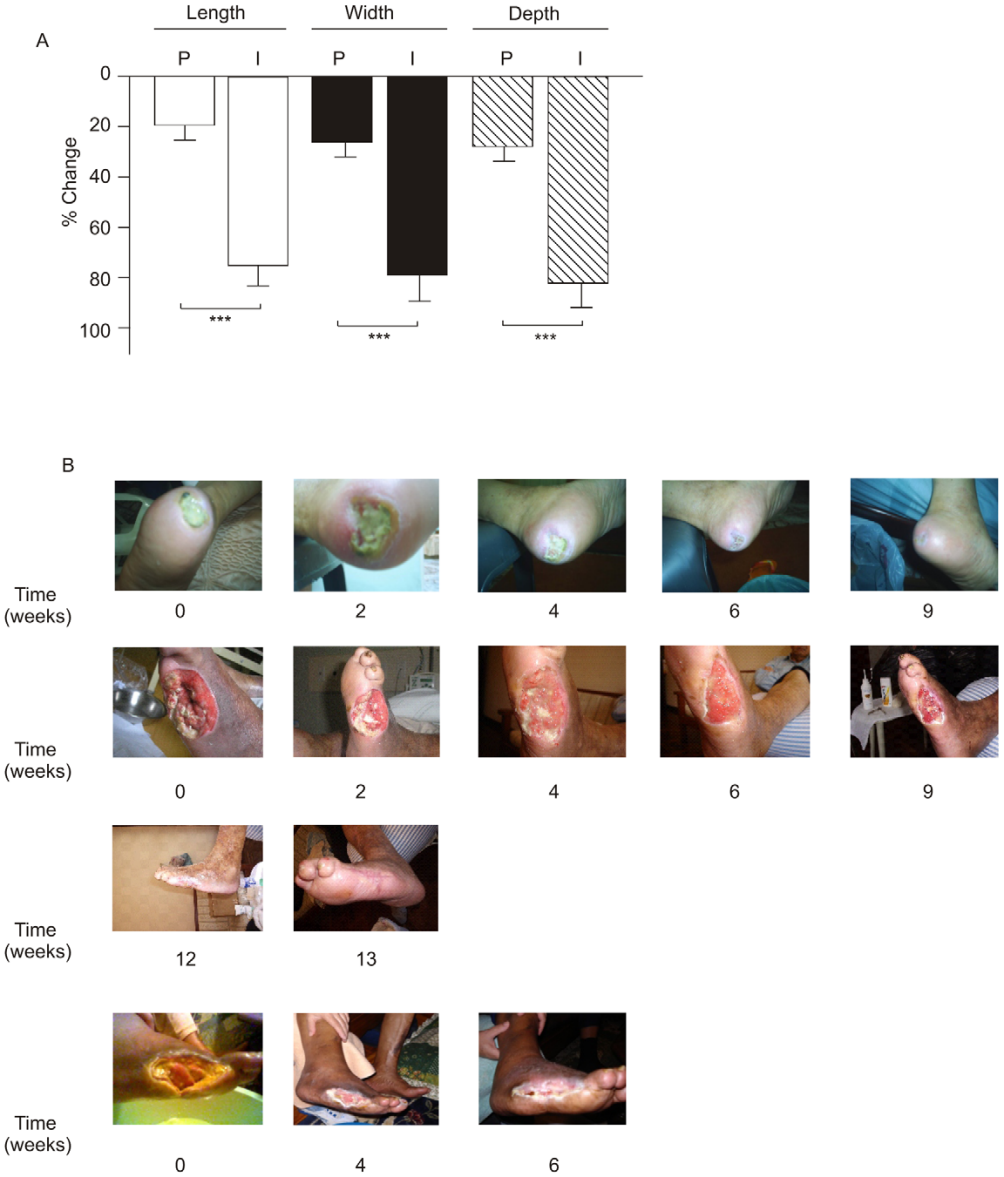
Percentage of change of ulcers in patients. (A) Change in length, width, and depth of ulcers in the two groups of diabetic patients that received the insulin cream (I) or placebo (P). (B) Photos showing the time-course of wound healing in patients 0, 2, 4, 6, 9, 12, and 13 weeks after receiving the insulin cream. *P<0.001 *vs.* placebo.

Twenty-two patients, eight females and 14 males, completed the eight-week study protocol (Fig. 1). The final outcome criterion in this study was the change in ulcer dimension within the eight weeks of follow-up. There were no significant differences in clinical data between patients in the two groups (Table 2). By the end of the 8th week, the 12 patients that received the placebo cream showed only a very mild improvement, while the 10 patients that used the insulin cream presented a significant improvement. The improvement of the wound healing after the treatment was obtained between eight and 15 weeks. One-way ANOVA showed a statistically significant difference among insulin cream and placebo with regard to the decrease in length (P<0.001), width (P<0.001), and depth (P<0.001) of the wound (Fig. 6A). Complete healing occurred in four patients in the insulin-cream group (I) and in no patients in the placebo group. Pictures of three patients in group I are shown in Figure 6B.

**Table 2:** Clinical and laboratory characteristics of diabetic patients, treated with placebo (P) or insulin cream (I).

Variable	N (%)	Mean (± SD)	
		P	I
Male	14 (63.64)		
Female	8 (36.36)		
Age (years)		64.0 (±10.5)	62.0 (±11.1)
Time since diagnosis of diabetes mellitus (years)		8.6 (±6.1)	9.9 (±7.7)
Body mass index {weight (kg)/height(m)^2^}		28.5 (±6.2)	29.7 (±4.9)
Glycosylated hemoglobin		8.1 (±2.8)	7.9 (±2.4)
Fasting glycemia		179.6 (±72.2)	170.8 (±66.3)
Creatinine		1.0 (±0.2)	0.9(±0.16)
Leukocytes		5600 (±9000)	5870 (±1.100)

There were no significant differences between the groups, according to a paired t-test.

Since there was an impressive improvement in wound healing in the patients of group I, we decided to offer the insulin cream to the patients of group P, after the eight weeks of placebo. In this regard our final analysis of time for complete healing included all the 22 patients (12 from the placebo group for eight weeks that changed to insulin, and 10 from the insulin group) that used the insulin cream until complete healing was achieved. Complete healing after initiating insulin cream occurred in seven patients at week 8, in three patients at week 9, in two patients at week 10, in four patients at week 12, in three patients at week 13, in two patients at week 14, and in three patients at week 15.

## Discussion

The results of the present study show that the insulin signaling pathways are upregulated in the wounded skin of normal rats, but in the wounded skin of diabetic animals these upregulations are blunted. However, when the wounded skin of diabetic rats is treated with a topical insulin cream, an acceleration of wound healing occurs, in association with a recovery in the proteins of the insulin signaling pathways [28].

Our data show that the expression of proteins involved in early steps of insulin action, i.e., IR/IRS-1,2/AKT, are increased in the healing tissue of wounds, compared to intact skin. AKT has the ability to phosphorylate proteins that regulate lipid synthesis, glycogen synthesis, cell survival, and protein synthesis [18], [29], [30]. Recently, data from different sources demonstrated that AKT activation is an important step for VEGF release in skin wounds, through a post-transcriptional mechanism in keratinocytes [31], [32], and is necessary for vascular maturation and angiogenesis during cutaneous wound healing [33]. Therefore, the increase in this signaling pathway observed in the healing skin of wounds might contribute to the process of tissue repair in skin. Insulin stimulation of ERK involves the tyrosine phosphorylation of IRS proteins and/or SHC, which in turn interact with the adapter protein, Grb2 (growth factor receptor-bound protein-2), recruiting the Son-of-sevenless (SOS) exchange protein to the plasma membrane for activation of Ras (one member of a large family of small molecular weight GTP-binding proteins) [34]. Once activated, Ras operates as a molecular switch, stimulating a serine kinase cascade through the stepwise activation of Raf, MEK (protein kinase that activates MAP kinases), and ERK. Activated ERK can translocate into the nucleus, where it catalyzes the phosphorylation of transcription factors, initiating a transcriptional program that leads to cellular proliferation or differentiation [35], [36]. Our results also show that protein levels of SHC and ERK are increased in the wounded skin, suggesting that the ERK signaling pathway can also play a direct pivotal role in the regulation of cellular growth and differentiation. It is important to emphasize that ERK activation is essential for keratinocyte pro-migratory signaling pathways [3], [27], [35], [37], [38], [39].

Furthermore, we observed that tissue expression of these proteins is attenuated in wounded skin of diabetic rats compared with the increase observed in wounded skin of control rats. Therefore, we can suggest that the abnormal insulin signaling observed in wounded skin of diabetic rats might contribute to the impaired wound healing observed as a complication of diabetes. There are probably several mechanisms that can attenuate insulin signaling in the wounded skin of the diabetic. First, it is known that elevated levels of glucose affect insulin signaling by regulating the expression of several genes, including the insulin receptor gene, at both the transcriptional and translational levels [40]. Moreover, hyperglycemia was shown to inhibit insulin action as a result of serine phosphorylation of IRS through a PKC-mediated mechanism, which may in turn increase the degradation of IRS proteins [41], [42].

In accordance with a downregulation of insulin signaling proteins in wound healing of diabetic animals, Goren et al. showed that insulin signaling proteins, including IRβ, IRS-1, IRS-2, and phosphorylated GSK3β were almost absent in acutely healing skin from ob/ob mice [43]. It is important to mention that in this type 2 diabetes obese animal model, leptin is absent and there is an increase in circulating TNFα. In this regard, this previous study showed that the administration of leptin or the infusion of anti-TNFα reversed the alterations in insulin signaling proteins and improved wound healing. Our data, by using a hypoinsulinemic animal model of diabetes showed that not only IR/IRSs/PI3k/Akt pathway but also the SHC/ERK pathway are downregulated in the wounded skin of diabetic animal. In addition, we show that the insulin cream can completely restore these alterations.

A previous study showed that diabetic rat serum stimulated collagen synthesis to a significantly lesser extent than normal rat serum [44]. On the other hand, topical use of insulin improves wound healing [7] and it is known that insulin stimulates [^3^H]thymidine incorporation into human skin fibroblasts [45], [46]. In addition, insulin strongly and specifically stimulates collagen synthesis in skin fibroblasts [44]. These data encouraged us to prepare a cream containing insulin, with the aim of accelerating wound healing in diabetes. Our data shows that the insulin cream normalizes the wound healing in the skin of diabetic rats and, in parallel, induces a recovery in the tissue level of all proteins involved in early steps of insulin action.

**Figure 7:**
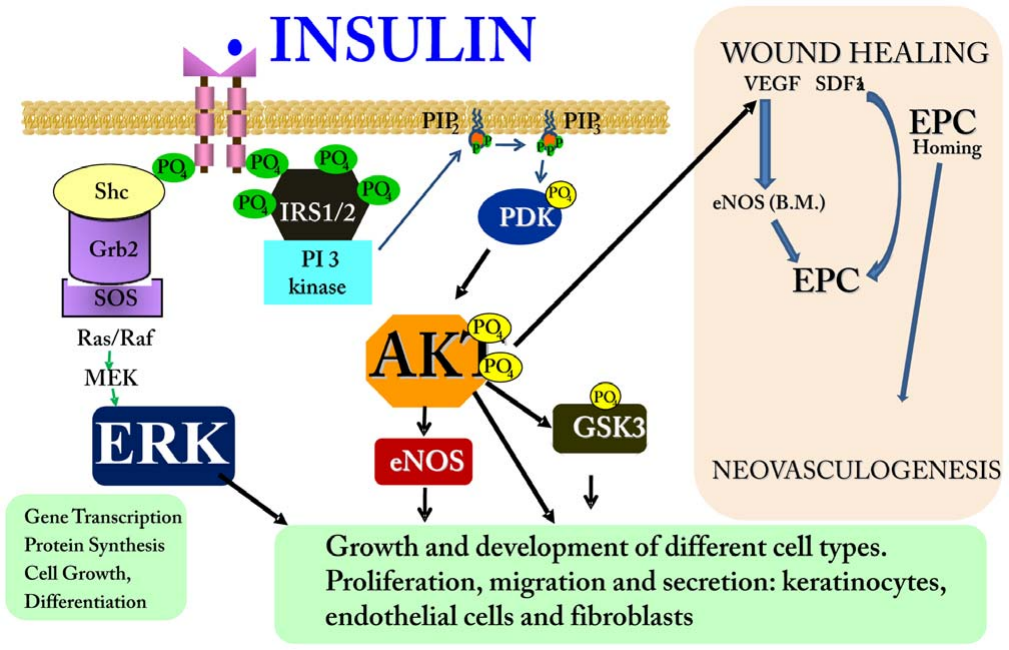
Effect of insulin on cellular and molecular mechanisms of wound healing in diabetes. Insulin induces activation of IR/SHC/ERK and IR/IRS/PI3K/AKT pathways in wound healing, which are canonical insulin signaling pathways. On the upper right-hand side, AKT is shown to increase VEGF (probably from macrophages, fibroblasts and epithelial cells) that will induce the phosphorylation and activation of eNOS in bone marrow, with consequent mobilization of EPCs to the circulation. SDF1α induces the homing of these EPC at the injury site, where they participate in neovasculogenesis. Insulin cream increased VEGF and SDF1α tissue expression in wound healing, and also increased eNOS phosphorylation in the bone marrow of an animal model of diabetes.

The molecular mechanisms by which insulin accelerates wound healing in diabetes seem to be many. The increase in proteins involved in the early steps of insulin action may play a role, since AKT and ERK have important growth and development effects. Additionally, the use of inhibitors of these pathways reduced the effect of insulin, suggesting that insulin uses both pathways to increase wound healing. At least two important substrates of AKT—GSK3β and eNOS—may have an important role in wound healing [30], [47]. GSK3β, when phosphorylated by AKT, has a reduced activity. It was recently demonstrated that mice harboring a fibroblast-specific GSK3β deficiency exhibit elevated collagen production, reduced apoptosis, and accelerated wound closure [47]. Thus, an increase in GSK3β phosphorylation, and a consequent reduction in its activity, may be one mechanism by which AKT can increase wound healing. AKT can also phosphorylate eNOS and promote NO production [48], [49], enhancing blood flow, cell survival, morphogenesis, and angiogenesis, even in the setting of ischemia [50], [51]. The multitude of AKT substrates and their described effects on various cellular functions may contribute, at least in part, to the beneficial effect of the insulin cream in wound healing, since this cream increases AKT protein expression and phosphorylation in the wounded skin of diabetic rats. Our data clearly show that the use of this insulin cream is an efficient manner to activate the AKT and ERK pathways, which are essential in the control of wound healing (Fig. 7).

It is now well established that an increase in the migration of EPCs from bone marrow to wounded skin accelerates wound healing. The regulation of this process is complex and involves activation of eNOS in the bone marrow by VEGF (produced in the wounded skin), enhancing the mobilization of EPC, which is recruited to the cutaneous wound site by an increase in tissue levels of SDF-1α [18], [19]. Our data, in accordance with results of a previous paper [19], showed that this complex process is downregulated in diabetic rats. However, interestingly, the use of an insulin cream in wounded skin, increased the tissue expression of VEGF, increased eNOS phosphorylation in the bone marrow, and increased SDF-1α in the wounded skin of diabetic animals. It is important to emphasize that the treatment of diabetic animals with subcutaneous insulin for one week was not able to restore eNOS phosphorylation or increase SDF-1α in the wounded skin of diabetic animals (data not shown).

In diabetic patients, growth factors are major technological advances that promise to change the face of wound healing [8], [52], [53], [54]. The most important growth factors used are recombinant human platelet-derived growth factor-BB (PDGF), granulocyte colony-stimulating factor (CSF), and epidermal growth factor. Many clinical trials have used these growth factors and shown only a mild improvement in wound healing [53], [54]. In addition, these growth factors are usually very expensive. Our results, with diabetic patients randomized to receive topical insulin or placebo in a prospective, double-blind and placebo-controlled clinical trial, show that the application of a cream containing insulin is able to significantly improve wound healing in these patients and, although the patients had very different sizes of ulcers, we observed complete healing at week 15 in all the 22 patients that used this cream. Previous pilot studies in animals or humans have employed topical insulin to accelerate wound healing in diabetes and, although these studies were not well designed, they all show an effect of insulin on this process [7], [8], [9], [10], [11], [12], [13], [14], [15], [55]. The insulin cream we made allowed us to prepare a homogenous cream, and improved the adherence of the cream to the surface of the wound. This product is practical and easy to use and, as demonstrated, is completely safe and did not induce hypoglycemia.

In contrast to other growth factors, insulin is much cheaper and available everywhere. Thus, with these results, we may suggest that a cream containing insulin is a cheaper and efficient adjunctive active wound therapy for diabetic patients.

In summary, our results show that tissue expression of IR, IRS-1, IRS-2, SHC, ERK, and AKT are increased in wound healing tissue, compared to intact skin, suggesting that the insulin signaling pathway may have an important role in wound healing. We also found that these pathways were attenuated in the wounded skin of diabetic rats, when compared to the wounded skin of normal rats, in parallel with an increase in the time for wound closure. Therefore, an insulin cream administered on the wound skin of diabetic animals, improved wound healing, and reversed the reductions observed in proteins of the insulin signaling pathways. In addition, the treatment also increased the expression of other proteins, such as eNOS (also in bone marrow), VEGF, and SDF-1α in wounded skin. In diabetic patients, this insulin cream was able to improve wound healing, offering a genuine, cheap and efficient treatment for this devastating complication of diabetes.

## Supporting Information

Approval S1 Approval by the Ethics Committee on animal study, State University of Campinas, São Paulo, Brazil.
(PDF)

Approval S2 Approval by the Ethics Committee on human study, State University of Campinas, São Paulo, Brazil.
(PDF)

Checklist S1 CONSORT checklist.
(DOC)

Protocol S1 Trial Protocol.
(DOC)
