# Expression of Concern: Topical Insulin Accelerates Wound Healing in Diabetes by Enhancing the AKT and ERK Pathways: A Double-Blind Placebo-Controlled Clinical Trial

**DOI:** 10.1371/journal.pone.0298558

**Published:** 2024-02-06

**Authors:** The PLOS ONE Editors

Following the publication of this article [1], concerns were raised regarding results presented in Figs. 2 and 4. Specifically,

- The same IB: Actin panel is shown in Figs. 2E and 2F.
- The Fig. 2G IB: Actin panel in this article [1] appears similar to the Figure 2E βactin panel of [2, 3] despite being used to represent different experimental conditions.
- The same IB: Actin panel is shown in Figs. 4E and 4F.

The corresponding author stated that the panel duplications within Fig. 2 and Fig. 4 were intentional: the same control blot was used for each pair of figure panels because the same amounts of the same tissue samples were used in the respective experiments. The corresponding author’s explanation appears to contradict the figure legends, which state that equal protein loading was confirmed by reblotting the membranes with anti-β-actin.

Some of the original data underlying Fig. 2 and Fig. 4 have been provided in the S1 and S2 Files provided with this notice. However, the authors stated that the original blots underlying the panels of concern are no longer available. In the absence of the original image data the concern about Fig. 2G cannot be resolved. In addition, the underlying data provided in the S2 File appear to be a processed data set as opposed to individual level data.

The *PLOS ONE* Editors issue this Expression of Concern to notify readers of the above concerns and to relay the available data provided by the corresponding author.

The Fig. 2G IB: Actin panel reports material from [2, 3], published in 2012 by the American Diabetes Association, which is offered under a CC-BY-NC-ND license, and are therefore excluded from this article’s [1] license. At the time of publication of this Expression of Concern notice, the article [1] was republished to note this exclusion in the Figure 2 legends and the article’s copyright statement.

## Supporting information

S1 File Available underlying blots for Figs. 2 and 4. (PDF)

S2 File Underlying data used to prepare the graphs presented in Figs. 2 and 4. (XLSX)
