# Supplementary material for: Topical Insulin Accelerates Wound Healing in Diabetes by Enhancing the AKT and ERK Pathways: A Double-Blind Placebo-Controlled Clinical Trial
Source: PLoS One. 2012 May 25;7(5):e36974. doi: 10.1371/journal.pone.0036974 (PMC3360697; doi:10.1371/journal.pone.0036974)
Supplement: Approval S1 — Approval by the Ethics Committee on animal study, State University of Campinas, São Paulo, Brazil. (PDF) [file pone.0036974.s001.pdf]

UNICAMP

CEEA/Unicamp

Comissão de Ética na Experimentação Animal
CEEA/Unicamp

CERTIFICADO

Certificamos que o Protocolo nº **1941-1**, sobre **"Efeito combinado de fenofibrato via oral e creme enriquecido com insulina tópico na cicatrização de lesões de animais diabéticos"**, sob a responsabilidade de **Profa. Dra. Maria Helena de Melo Lima / Lélia Lelis Ferreira de Abreu**, está de acordo com os Princípios Éticos na Experimentação Animal adotados pelo Colégio Brasileiro de Experimentação Animal (COBEA), tendo sido aprovado pela Comissão de Ética na Experimentação Animal – CEEA/Unicamp em **31 de agosto de 2009**.

CERTIFICATE

We certify that the protocol nº **1941-1**, entitled **"Combined effetc of oral fenofibrate and topical cream enriched with insulin in wound healing in diabetic animals"**, is in agreement with the Ethical Principles for Animal Research established by the Brazilian College for Animal Experimentation (COBEA). This project was approved by the institutional Committee for Ethics in Animal Research (State University of Campinas - Unicamp) on **August 31, 2009**.

Campinas, 31 de agosto de 2009.

Profa. Dra. Ana Maria A. Guaraldo
Presidente

Fátima Alonso
Secretária Executiva

CEEA – Unicamp
Caixa Postal 6109
13083-970 Campinas, SP – Brasil

Telefone: (19) 3521-6359
E-mail: comisib@unicamp.br
http://www.ib.unicamp.br/ceea/
