# Supplementary material for: Topical Insulin Accelerates Wound Healing in Diabetes by Enhancing the AKT and ERK Pathways: A Double-Blind Placebo-Controlled Clinical Trial
Source: PLoS One. 2012 May 25;7(5):e36974. doi: 10.1371/journal.pone.0036974 (PMC3360697; doi:10.1371/journal.pone.0036974)
Supplement: Approval S2 — Approval by the Ethics Committee on human study, State University of Campinas, São Paulo, Brazil. (PDF) [file pone.0036974.s002.pdf]

FACULDADE DE CIÊNCIAS MÉDICAS
COMITÊ DE ÉTICA EM PESQUISA
Caixa Postal 6111, 13083-970 Campinas, SP
(0_19) 3788-8936
FAX (0_19) 3788-8925
www.fcm.unicamp.br/pesquisa/etica/index.html
cep@fcm.unicamp.br

CEP, 20/01/04.
(Grupo III)

PARECER PROJETO: N° 652/2003

I-IDENTIFICAÇÃO:

PROJETO: **"EFEITO DA INSULINA NA CICATRIZAÇÃO DE FERIDAS EM PACIENTES DIABÉTICOS".**
PESQUISADOR RESPONSÁVEL: Maria Helena de Melo Lima
INSTITUIÇÃO: HC/UNICAMP
APRESENTAÇÃO AO CEP: 19/12/2004
APRESENTAR RELATÓRIO EM: 20/01/05

II - OBJETIVOS

Verificar a incidência de úlceras e se a pasta de insulina é eficaz na cicatrização de feridas não-infectadas de diabéticos tipo 2.

III - SUMÁRIO

Serão avaliados 40 portadores de diabetes tipo 2 e divididos em dois grupos.Em um grupo estarão os pacientes com múltiplas úlceras. Nesse grupo, uma parte das úlceras do mesmo indivíduo será tratada com pasta de insulina e as restantes com o veículo. No segundo grupo, que são aqueles indivíduos que apresentam lesão única, metade dos indivíduos serão tratados com pasta de insulina e metade com veículo.

IV - COMENTÁRIOS DOS RELATORES

Trata-se de um projeto que irá avaliar a utilização de pasta de insulina na cicatrização de feridas de diabéticos. O projeto está de acordo com as resoluções vigentes. O Termo de Consentimento Livre e Esclarecido é simple mas adequado para o tipo de estudo.

V - PARECER DO CEP

O Comitê de Ética em Pesquisa da Faculdade de Ciências Médicas da UNICAMP, após acatar os pareceres dos membros-relatores previamente designados para o presente caso e atendendo todos os dispositivos das Resoluções 196/96 e complementares, bem como ter aprovado o Termo do Consentimento Livre e Esclarecido, assim como todos os anexos incluídos na Pesquisa, resolve aprovar sem restrições o Protocolo de Pesquisa supracitado.

O conteúdo e as conclusões aqui apresentados são de responsabilidade exclusiva do CEP/FCM/UNICAMP e não representam a opinião da Universidade Estadual de Campinas nem a comprometem.

VI - INFORMAÇÕES COMPLEMENTARES

O sujeito da pesquisa tem a liberdade de recusar-se a participar ou de retirar seu consentimento em qualquer fase da pesquisa, sem penalização alguma e sem prejuízo ao seu cuidado (Res. CNS 196/96 – Item IV.1.f) e deve receber uma cópia do Termo de Consentimento Livre e Esclarecido, na íntegra, por ele assinado (Item IV.2.d).

Pesquisador deve desenvolver a pesquisa conforme delineada no protocolo aprovado e descontinuar o estudo somente após análise das razões da descontinuidade pelo CEP que o aprovou (Res. CNS Item III.1.z), exceto quando perceber risco ou dano não previsto ao sujeito participante ou quando constatar a superioridade do regime oferecido a um dos grupos de pesquisa (Item V.3.).

O CEP deve ser informado de todos os efeitos adversos ou fatos relevantes que alterem o curso normal do estudo (Res. CNS Item V.4.). É papel do pesquisador assegurar medidas imediatas adequadas frente a evento adverso grave ocorrido (mesmo que tenha sido em outro centro) e enviar notificação ao CEP e à Agência Nacional de Vigilância Sanitária – ANVISA – junto com seu posicionamento.

Eventuais modificações ou emendas ao protocolo devem ser apresentadas ao CEP de forma clara e sucinta, identificando a parte do protocolo a ser modificada e suas justificativas. Em caso de projeto do Grupo I ou II apresentados anteriormente à ANVISA, o pesquisador ou patrocinador deve enviá-las também à mesma junto com o parecer aprovatório do CEP, para serem juntadas ao protocolo inicial (Res. 251/97, Item III.2.e)

Relatórios parciais e final devem ser apresentados ao CEP, de acordo com os prazos estabelecidos na Resolução CNS-MS 196/96.

VII - DATA DA REUNIÃO

Homologado na I Reunião Ordinária do CEP/FCM, em 20 de janeiro de 2004.

Profa. Dra. Carmen Sílvia Bertuzzo
PRESIDENTE DO COMITÊ DE ÉTICA EM PESQUISA
FCM / UNICAMP
