# Supplementary material for: Topical Insulin Accelerates Wound Healing in Diabetes by Enhancing the AKT and ERK Pathways: A Double-Blind Placebo-Controlled Clinical Trial
Source: PLoS One. 2012 May 25;7(5):e36974. doi: 10.1371/journal.pone.0036974 (PMC3360697; doi:10.1371/journal.pone.0036974)
Supplement: Protocol S1 — Trial Protocol. (DOC) [file pone.0036974.s004.doc]

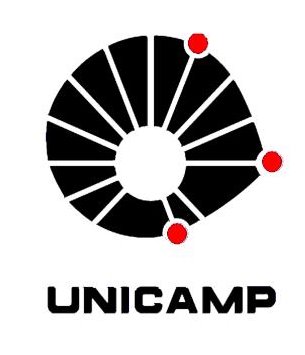


A Randomized, Double-blind, Placebo-controlled Study Concerning the Effect of the Insulin Cream on the Wound Healing of Diabetic Patients

Principal Investigator: Mario J.A. Saad

# Co-investigators: Maria Helena M. Lima, Andréa M. Caricilli, Lélia L. de Abreu, Eliana P. Araújo, Fabiana F. Pelegrinelli, Ana C. P Thirone, Daniela M. Tsukumo, Aparecida de Moraes, José B. C. Carvalheira, Lício A. Velloso.

Mailing Address: Rua Tessália Vieira de Camargo, 126, Cidade Universitária “Zeferino Vaz” – Campinas/SP – Brazil – CEP: 13083-887

P.I.’s Telephone: +55 19 35218950

**Study Protocol**

**(Purpose of the study)**

The purpose of this study is to determine whether topic insulin is effective to accelerate wound healing in diabetic patients.

**(Background of the study)**

An indolent, non-healing wound constitutes a major problem that plagues those with diabetes. Approximately 15% of all patients with diabetes will, at some time, have non-healing wounds, despite insulin treatment and a meticulously-controlled diet, and this is the leading cause of lower extremity amputation. However, the available treatments are awfully expensive or not effective enough.

**(Methods)**

1. The study design

This is a randomized, double-blind, placebo-controlled study. The patients will be recruited in the University Hospital of the State University of Campinas. The trial will be explained to all patients, and their written informed consent will be obtained before any trial-related procedures initiates.

2. Eligibility

Eligible patients are 18 to 80 years-old, both male and female patients with type 1 or type 2 diabetes mellitus with established diagnosis for more than 2 years, and with wounds that were difficult to be healed for at least three months. The probands should also have serum creatinine below 1.5mg/dL.

3. Exclusion criteria

Patients will be excluded if they do not satisfy the inclusion criteria, if the wounds are infected, if cellulites, venous stasis, inadequate perfusion and osteomyelitis are present, and if the patients are unable to attend the follow-up.

4. Informed consent

Patients who are eligible and not excluded according to the exclusion criteria described above will be informed of the background, purpose, method, spontaneous participation, withdraw rights of the study, and gave written consent. Study subjects can read this protocol if necessary.

5. Interventions

Patients who meet the criteria and seen between January 2004 and September 2007 will be considered for the study.

Group I intervention will consist of debridement for the ulcer, administration of the insulin cream (patent number: PI 0705370-3, University of Campinas, Brazil) on the wound, digital photograph by the same observer with the same camera (Sony, Cybershot, 8.1 megapixels) and measurement of the wound (height (↕) x width (↔) weekly, during 8-12 weeks. Group P intervention will consist of debridement for the ulcer, administration of the placebo cream on the wound, digital photograph by the same observer with the same camera (Sony, Cybershot, 8.1 megapixels) and measurement of the wound (height (↕) x width (↔) weekly, during 8-12 weeks.

Blood will be collected in both groups, in order to perform laboratory analysis, such as leukocyte count, hemoglobin, platelet count, glucose, creatinine, and glycosylated hemoglobin. These analyses will be made in the University Hospital of State University of Campinas, in the routine laboratory.

The detailed schedule is shown in the Table 1

Table 1. Schedule of the study

| Visit | Blood Sample | Digital  Photograph | Size of the wound height (↕) x width (↔) | Group I | Group P |
| --- | --- | --- | --- | --- | --- |
| 1 |  |  |  | Active | Placebo |
| 2 |  |  |  | Active | Placebo |
| 3 |  |  |  | Active | Placebo |
| 4 |  |  |  | Active | Placebo |
| 5 |  |  |  | Active | Placebo |
| 6 |  |  |  | Active | Placebo |
| 7 |  |  |  | Active | Placebo |
| 8 |  |  |  | Active | Placebo |

6. Target sample size

Target sample size is 46, which corresponds to the number of patients that attended the inclusion criteria.

7. Administration of the investigatory product (IP)

The insulin or the placebo cream will be administrated according to the schedule table shown in Table 1.

8. Investigatory product

The insulin cream used is prepared with regular insulin (0.5U/g cream) in the pharmacy of our University Hospital and holds the patent number, PI 0705370-3 (University of Campinas, Brazil). In preliminary experiments, we used different concentrations of insulin to prepare the cream, but the doses that induced the best effect in wound healing without changing blood glucose was 0.5U/g. The cream under study, vehicle or with insulin, is applied locally to cover the excision.

9. Randomization

Eligible participants will be provided unique subject identification numbers according to study criteria and will be assigned to the insulin cream group or to the placebo group by a research technician (L.L.A.), according to a computer-generated, randomization plan.

10. Limitation and fixation of concomitant medication

The oral drugs or s.c. insulin used by the patients to treat diabetes will not be changed during the treatment period with placebo cream or insulin cream, that will be at least 8 weeks.

11. Analysis

Primary outcome measure:

% of Wound healing closing measured each week [Time Frame: 7 days ] [ Designated as safety issue: Yes ].

Secondary outcome measure:

Complete wound healing [Time Frame: 8 weeks] [Designated as safety issue: Yes ]

Safety analysis

The prevalence of adverse events will be compared between the insulin and the placebo cream interventions.

12. Statistical analysis

Wound dimensions will be calculated in a blinded fashion and analyzed for homogeneity and significance using the SPSS, version 13.0 (SPSS, Inc., Chicago, IL, USA). All continuous variables will be expressed as means ± SE. One-way analysis of variance (ANOVA) will be used to assess the differences in a continuous variable between the two groups of patients. Post hoc analysis was performed using Tukey’s test. All tests will be two tailed, and the level of significance employed will be *P*<0.05.

[Response to adverse events]

In case of adverse events escalation of the investigatory product should be discarded, the investigatory product should be de-escalated or ceased if necessary. Adverse events should be reported to the principal investigator and the study supervisor.

[Handling of study data]

Data will be collected and fixed by the principal investigator. Data will be analyzed after key-open.

[Registration of the study]

The study will be registered in ClinicalTrials.gov.

[Publication of the study]

The study will be published to the journal under the names of the investigators,

or the name of study group if necessary.

ADDENDUM 1.

The form that will be fulfilled in the hospital follows the model below:

Name:_____________________________________________ Number:_____

Birthday date:­­­­­­­­­­­­­­­­___________________________________________________

Gender: □ Male □ Female

□ Type 1 diabetes □Type 2 diabetes Time after the diagnosis:_________

Time of wound:_________________ Wound location:____________________

Wound size:___________________ cm

Signs of infections: □ Yes □ No

Cellulitis: □ Yes □ No

Inadequate perfusion: □ Yes □ No

Osteomyelitis: □ Yes □ No

Beginning of the treatment:__/__/____ □ Insulin Cream □ Placebo

Serum creatinine:________________mg/dL

Weekly evaluation:

__/__/____ Wound size area:_______________cm (height (↕) x weidth(↔))

Aspect of the wound:________________________

Signs of infections: □ Yes □ No

Cellulitis: □ Yes □ No

Inadequate perfusion: □ Yes □ No

Osteomyelitis: □ Yes □ No

Weekly reduction of the wound area: ___________cm (height (↕) x weidth(↔))

Have photographs been taken? □ Yes □ No

ADDENDUM 2.

The consent form follows the model above:

**Effect of topical insulin on the wound healing of diabetic patients**

Name: _________________________________________ Birth date:__/__/__

Address: _______________________________________________________________

Phone number: ____________________________

Sponsor:________________________ ID:________________ Kinship:_____________

**Introduction**

This consent form might contain words which you may not understand. Ask the doctor of the study team any word or information you do not understand. Do not sign this consent for if you did not receive enough information.

**Aims and description of the study**

As a patient that presents diabetes or obesity, diseases that impair the healing process, you are invited to participate of this research. The aim of using this insulin cream in the surgery incision is to accelerate the wound healing.

**Proceedings of the study**

In the first visit, you will be evaluated by the doctor or by a person of the study team, in order to determine if you are able to participate of the study. To be eligible to participate of the study, you must be an obese or diabetic patient who has recently been submitted to surgery. After the initial visit, you must attend the reference service for a month, weekly, where your incision will be evaluated and you will receive the orientation of how to make the bandage using the insulin cream.

During all your visits, a member of the study team will ask questions about how you have felt since your last visit. You will also receive information about the bandage and the evolution of the wound.

**Risks**

The drug used in this study can only be used (topical use) by the person who has been prescribed to and should be kept away from children or people with limited ability of reading and understanding.

**Benefits of the treatment**

It is possible that you may not obtain any benefits with your participation in this study. However, you may have benefits with the treatment if the wound healing is accelerated (that is, the improvement of the healing, “closing” the incision faster).

**Participation**

Your participation in this study is voluntary. The quality of the medical treatment that you receive will not be affected in case you decide not to participate of this study. If you decide to participate of it, you will be able to leave it any moment, without any prejudice of the medical service or other penalties. Nevertheless, you can only participate of the study if your current intention is to proceed until the end of it (approximately one month). If the drug used in the study is suspended, you must continue attending the visits of the study for your condition to be evaluated. If you leave the study (that it, if you stop using the drug and attending the hospital), you must agree that the data about you general health condition keep being collected.

If you want to leave this study, talk to one of the researchers.

If you have any question about your rights as a participant of the study, call the Ethics Committee of Research: +55 19 35218936.

**Consent**

Signing this form, I declare that I have read and understood its content and have asked and received enough answers to all my doubts about this investigation, and also that I agree to participate of it.

I understand that I will receive a copy of this consent form.

**Signatures**

_______________________________________________

Name of the participant

_______________________________________________

Signature of the participant or sponsor

_______________________________________________

Name of the person who presented the consent

_______________________________________________

Signature of the person who presented the consent

I and/or my representative have discussed the nature and aims of this study, as well as the risks and benefits of the participation of it, with the participant or sponsor. I believe that the participant and/or the sponsor have received all the necessary information, which was provided in suitable and understandable language, and that s/he has understood it.

______________________________________________ ____________

Signature of the principal investigator Date

ADDENDUM 3.

The study was conducted in accordance with the guidelines of the University of Campinas for clinical trials and the Declaration of Helsinki. We have also obtained ethics approval for the human study from the University Hospital of the State University of Campinas (ID: 652/2003), where participants were recruited and human experimentation was conducted.
