# Supplementary figures and images for: Expression of Concern: Topical Insulin Accelerates Wound Healing in Diabetes by Enhancing the AKT and ERK Pathways: A Double-Blind Placebo-Controlled Clinical Trial
Source: PLoS One. 2024 Feb 6;19(2):e0298558. doi: 10.1371/journal.pone.0298558 (PMC10846732; doi:10.1371/journal.pone.0298558)

Fig 2A.

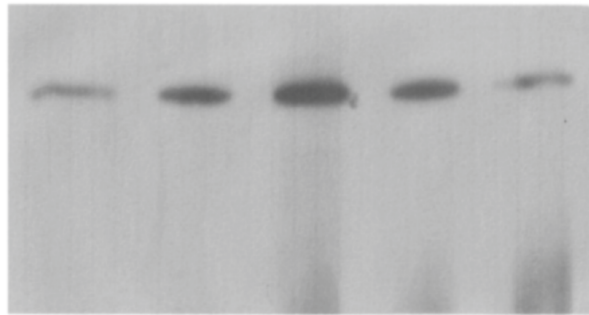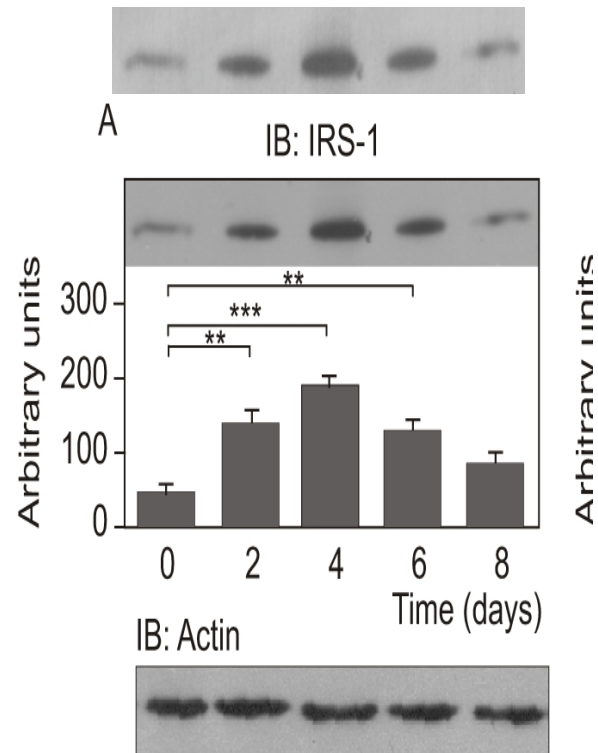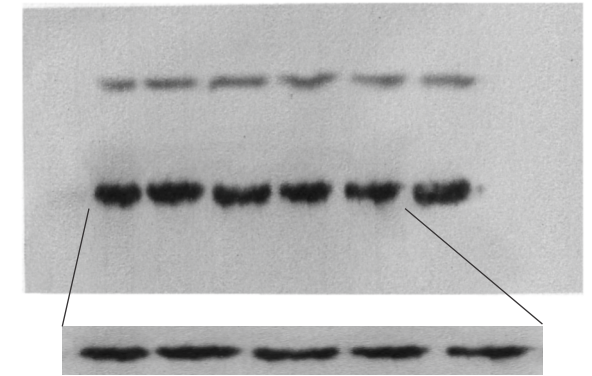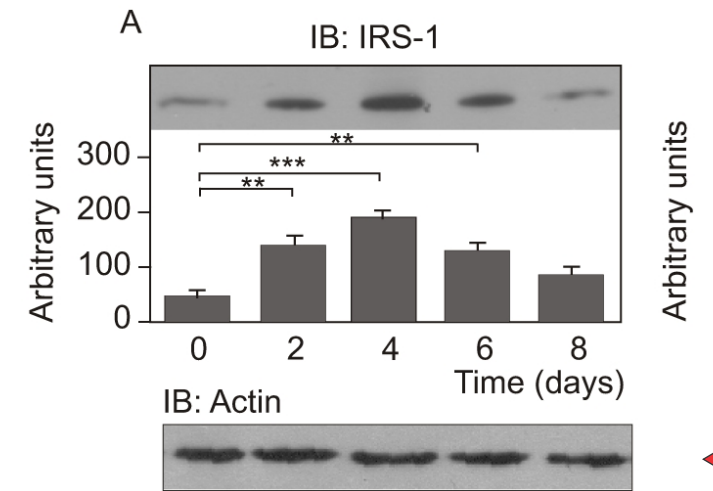

Fig 2B. AKT

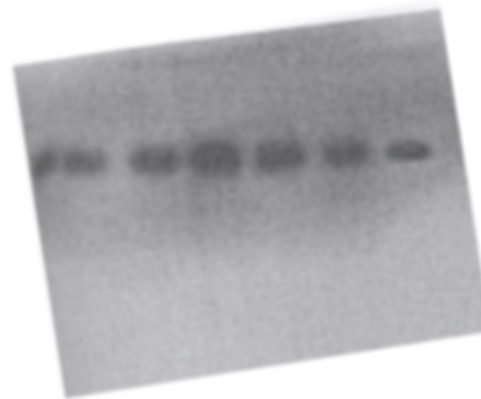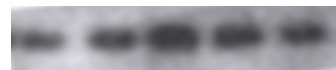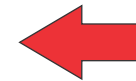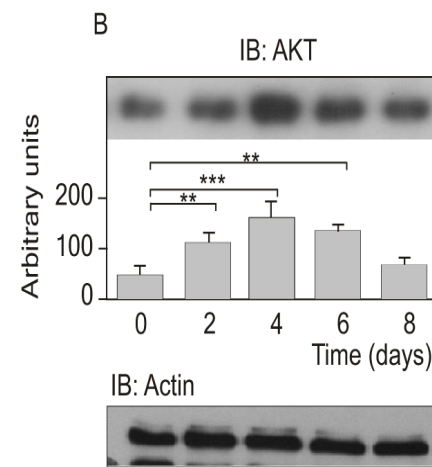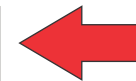

Fig 2e. IR

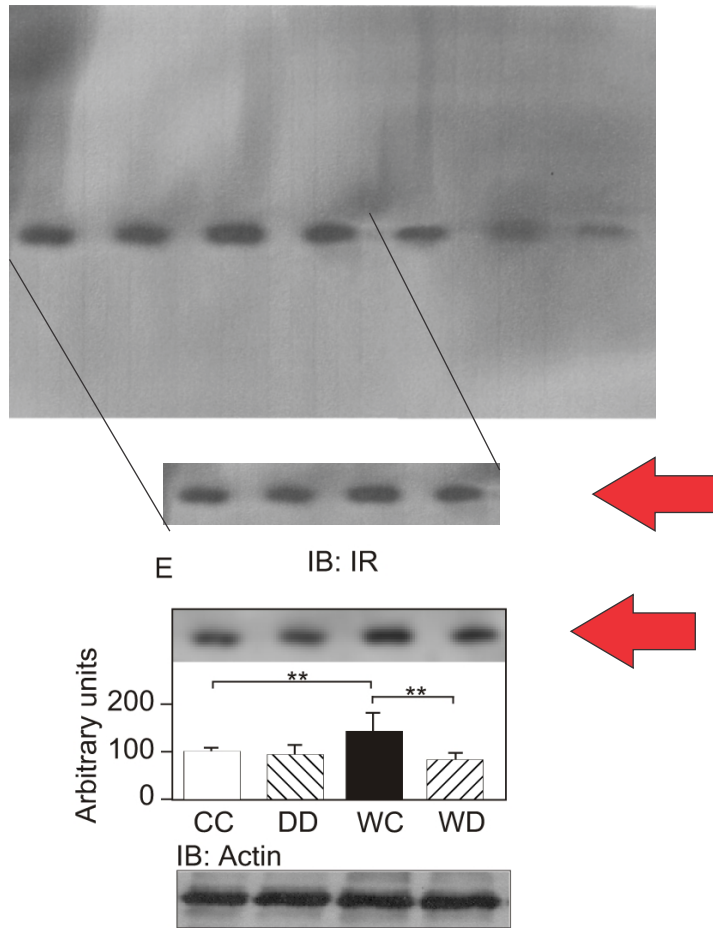

Fig 2I. AKT

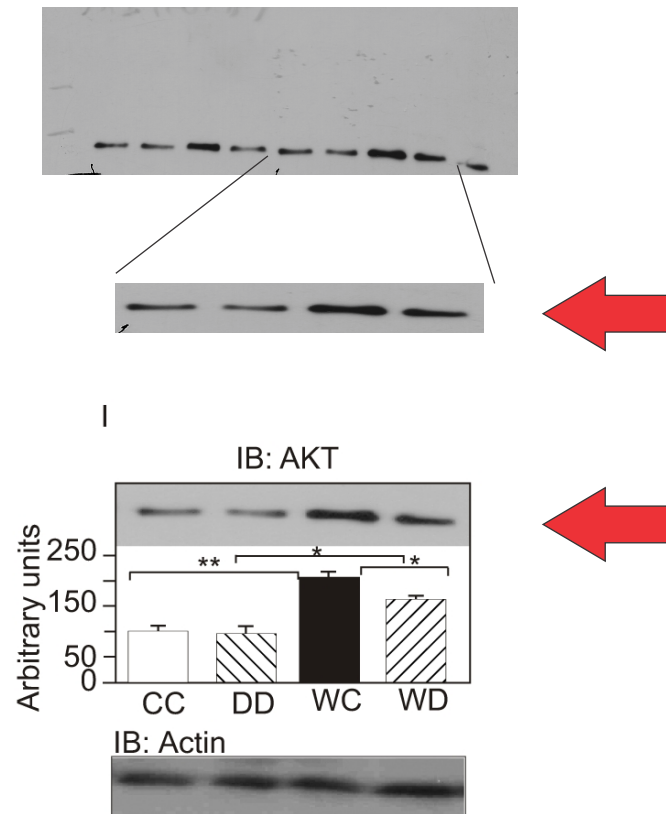

Fig 2J. ERK1/2

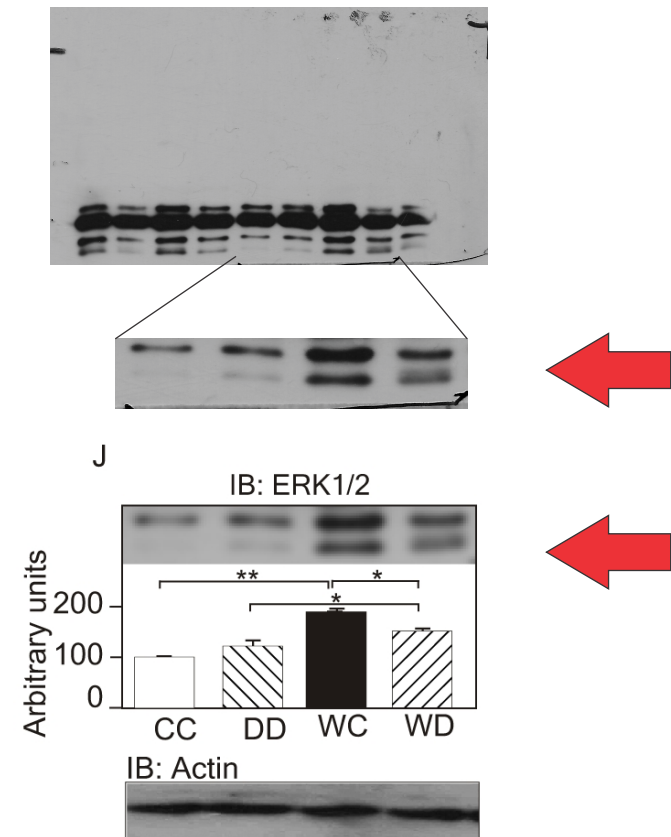

Fig 4C. IRS 1

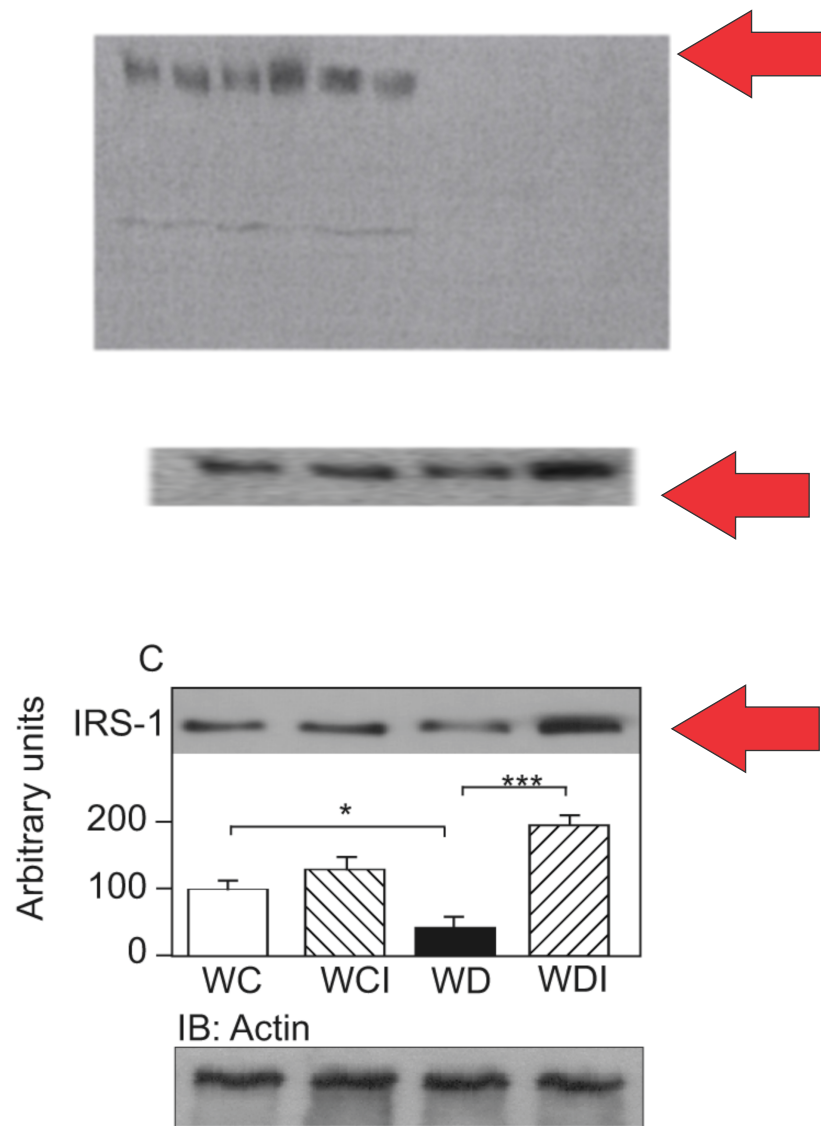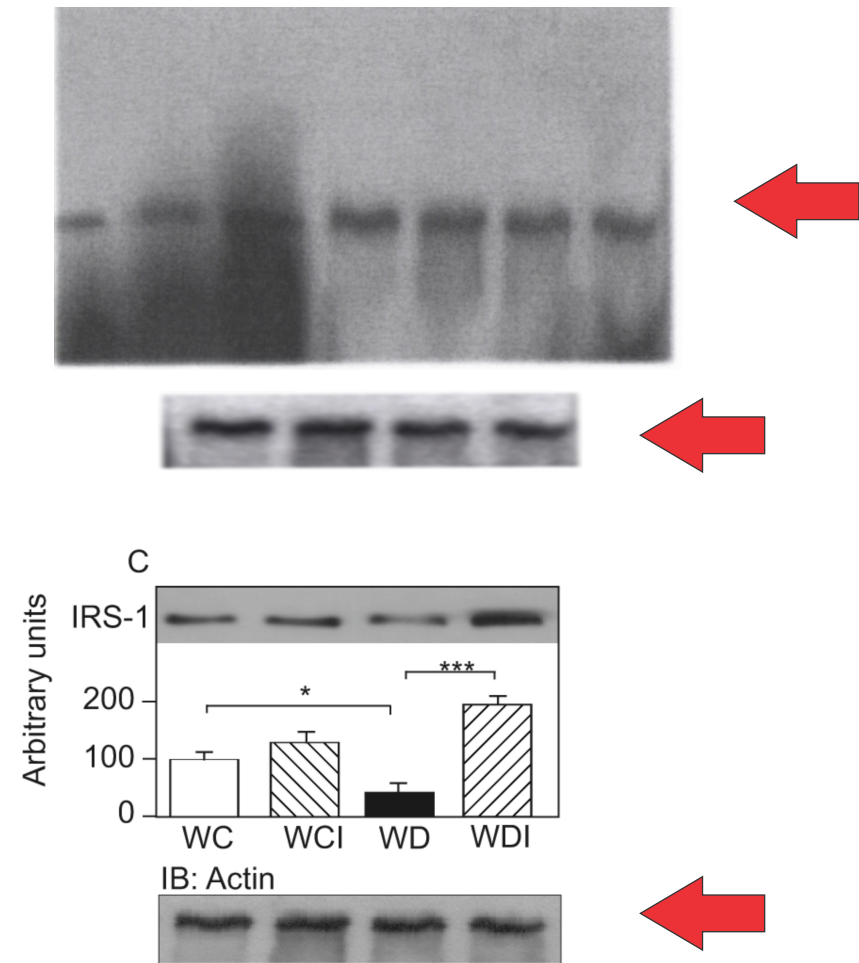

Fig 4D. Actin

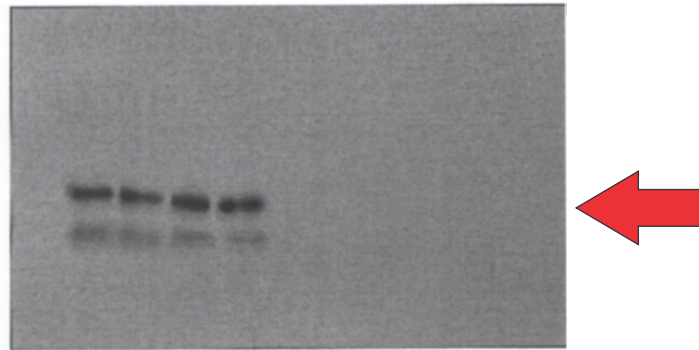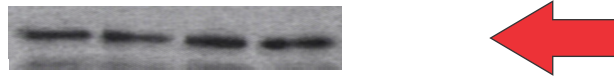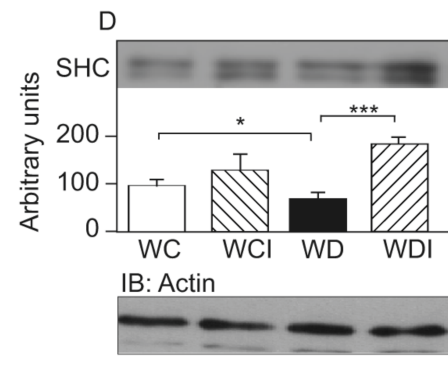

Fig 4H. eNOS

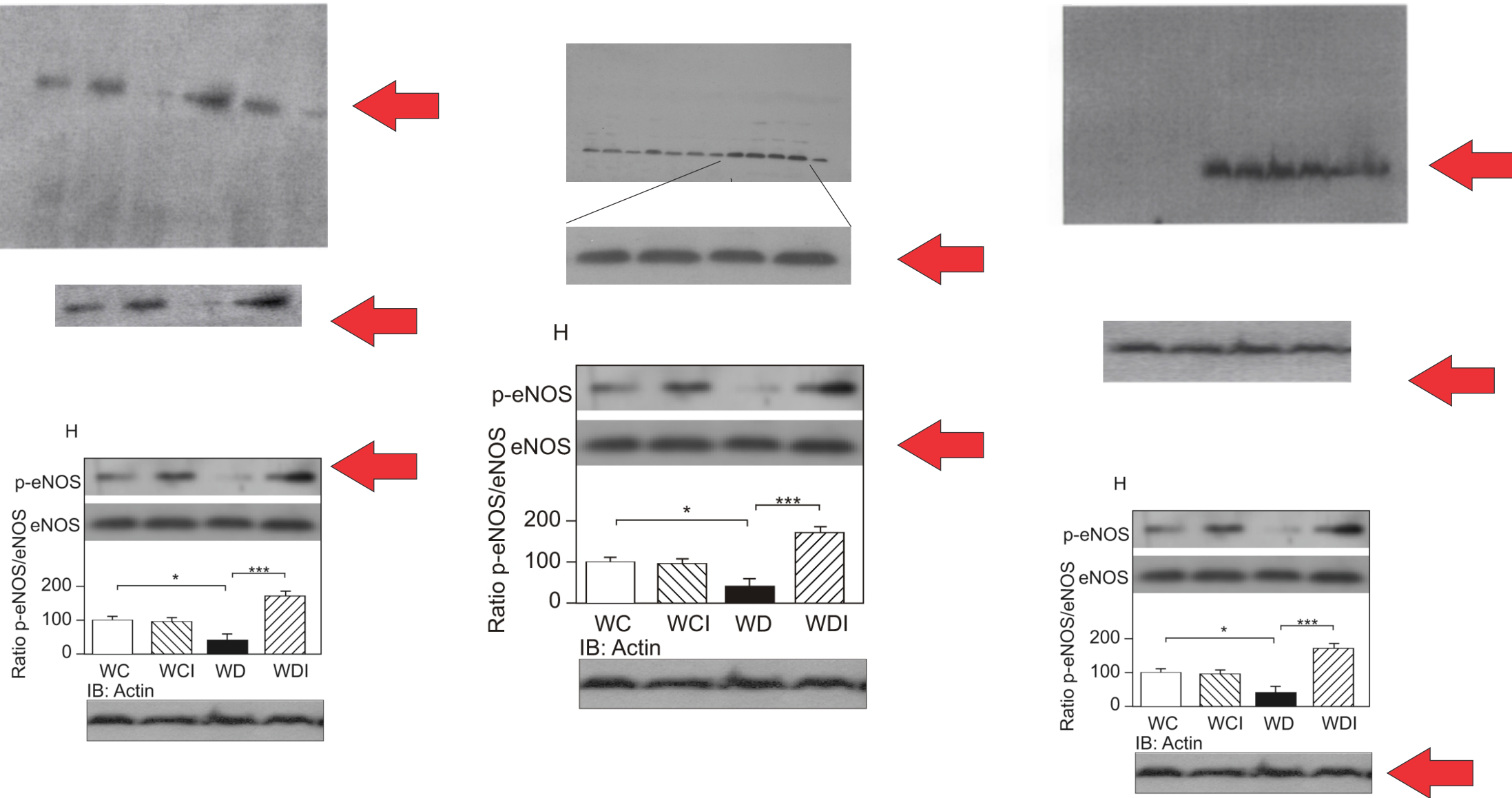

Fig 4G. GSK3

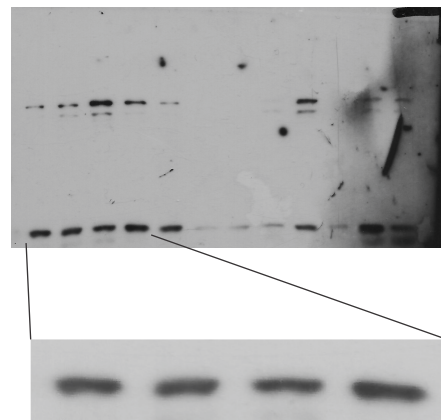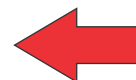

G

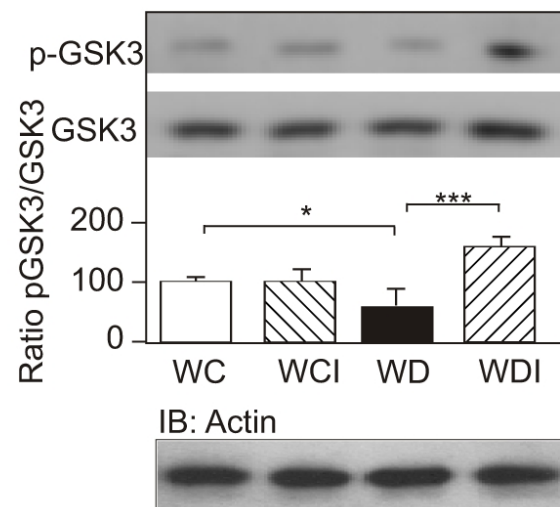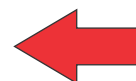

Supplement: S1 File — (PDF) [file pone.0298558.s001.pdf]
